# Compassion Questionnaires Revised: Scales Development and Validation

**DOI:** 10.1177/10731911251337185

**Published:** 2025-05-17

**Authors:** Bassam Khoury, Rodrigo C. Vergara

**Affiliations:** 1 McGill University, Montreal, QC, Canada; 2 Universidad Metropolitana de Ciencias de la Educación, Santiago, Chile; 3 Centro Nacional de Inteligencia Artificial CENIA, Macul, Chile

**Keywords:** compassion, self-compassion, skills, scale, questionnaire

## Abstract

The Compassion Questionnaires for Self and Others were developed to measure compassion as a multifaceted construct encompassing affective, cognitive, behavioral, and interpersonal dimensions. However, the original versions had limitations such as item number and coverage of the underlying concepts, unidirectional item wording, lack of a global latent variable, and validation only among women. This study aimed to address these shortcomings by revising the questionnaires to improve their psychometric properties. The revised Compassion Questionnaires for Self and Others underwent significant modifications. A large-scale validation study involving both women and non-women participants was conducted to evaluate the revised questionnaires. The final versions of the revised compassion questionnaires comprised 39 items for self-compassion and 33 items for compassion toward others, incorporating both positive and negative wording. Psychometric analysis indicated excellent reliability and validity, with evidence supporting the existence of global latent variables. The revised questionnaires represent a significant improvement over the original versions, offering a comprehensive operationalization of compassion constructs suitable for diverse populations. The study findings underscore the theoretical and practical significance of these questionnaires in assessing and cultivating compassion. However, certain limitations warrant consideration, and the implications for research and clinical practice are thoroughly discussed.

## Introduction

With a history extending over several centuries, the concept of compassion has played a pivotal role in diverse philosophical and religious traditions, most notably Buddhism (Shonin et al., 2014). In the last few decades, there has been a notable increase in the exploration of compassion and its implications for health and social well-being within the realm of Western psychological research. However, despite the growing interest in compassion, there has been no consensus on its definition or operationalization. Various researchers have defined compassion in different ways. Gilbert (2009) described it as a deep awareness of another’s suffering combined with a desire to help. Wispé (1991) added that compassion also involves a nonjudgmental attitude toward others. Neff (2003a) focused on self-compassion, defining it as having three key aspects: kindness toward oneself, mindfulness of one’s distress, and recognizing one’s suffering as a shared human experience. Similarly, Kanov et al. (2004) identified three core elements of compassion: noticing suffering, feeling empathy, and responding to alleviate it. In a review, Strauss et al. (2016) outlined a five-factor model of compassion: (a) Recognizing suffering; (b) Understanding that suffering is a universal human experience; (c) Feeling empathy and connecting emotionally with the person in distress; (d) Tolerating and accepting the uncomfortable feelings that arise in response to another’s suffering; and (e) Being motivated to act and help relieve the suffering.

Some of these conceptualizations have focused exclusively on self-compassion or compassion for others, while others have aimed to address both constructs. However, even when both constructs were considered, these conceptualizations did not always aim to directly interconnect them, leading to mixed results in the relationships between measures of compassion and self-compassion (as detailed below). This approach is not well aligned with Buddhist traditions, which view compassion toward oneself and others as highly interconnected (Crosby & Skilton, 1998; Maitreya et al., 2018), both conceptually and practically, with the cultivation of one fostering the other.

Based on these conceptualizations, different measures of compassion for self and others were developed and validated. Among these measures are the Self-Compassion Scale (SCS; Neff, 2003a) and the Compassion Scale (toward others) (CS; Pommier et al., 2020). Both scales are based on Neff’s conceptualization of self-compassion (Neff, 2003b) and comprise three dimensions, along with their negative counterparts. Self-kindness versus self-judgment reflects being caring and understanding toward oneself rather than being critical and judgmental. Common humanity versus isolation involves recognizing suffering as part of the shared human experience rather than feeling isolated or disconnected from others. Mindfulness versus over-identification focuses on being able to observe and acknowledge one’s thoughts and emotions with openness rather than being overly identified with or attached to them. While SCS uses the six factors detailed above, the CS uses only three of them: kindness, common humanity, and mindfulness.

Besides the compassion scales based on Neff’s conceptualization, Gilbert developed and validated the Compassionate Engagement and Action Scales for Self and Others (CEAS; Gilbert et al., 2017), which include recognizing distress in self or others while directing feelings of warmth and care toward self or others and engaging in helpful actions. In addition, based on Strauss et al. (2016) five-factor model for compassion (described above), the Sussex-Oxford Compassion Scale was developed and validated (SOCS; Gu et al., 2020). The SOCS assesses compassion for self (SOCSS) and compassion for others (SOCSO) through a 20-item self-report questionnaire. For detailed reviews of the conceptualizations and psychometric properties of existing compassion measures (see Khoury, 2019; Khoury & Dionne, 2020; Strauss et al., 2016).

A key limitation in these conceptualizations of compassion is that they often include mindfulness or elements of mindfulness, such as awareness or noticing, which can confuse the two concepts and lead to concept contamination (Boateng et al., 2018; Messick, 1995). Although mindfulness and compassion are related (Tirch, 2010), it is important for both research and clinical practice to clearly distinguish between them. In addition, separating the understanding of universal human suffering from other aspects of cognition, such as nonjudgment, raises questions and lacks clear justification in previous definitions (Khoury, 2019; Khoury & Dionne, 2020). Furthermore, these definitions do not directly connect to compassion-based interventions or the specific mechanisms that can be targeted in these interventions. Comprehensive reviews of existing literature on compassion, whether directed toward oneself or others, revealed an interplay of affective, cognitive, and interpersonal elements in the process of cultivating compassion (Khoury, 2019; Khoury & Dionne, 2020). Neuroscientific research investigating compassion training further supports the involvement of affective, cognitive, and motivational skills/abilities at various stages (for an extensive review, see Dahl et al., 2016).

This new conceptualization of compassion for self and others addresses the limitations of previous definitions by (a) removing mindfulness or its elements from the conceptualization and operationalization of compassion; (b) defining the interpersonal aspect of compassion independently from common humanity, anchoring it in the ability to connect with and receive/provide care to others, as common humanity has not received empirical support; and (c) facilitating the integration of compassion training into existing cognitive-behavioral treatments. In addition, this conceptualization offers a parallel between compassion for self and others, defining compassion as a set of straightforward skills and abilities that can be directly incorporated into existing mindfulness and compassion-based interventions.

Based on this conceptualization, two parallel (i.e., similarly structured) questionnaires: the Compassion Questionnaire towards the Self (CQS) and the Compassion Questionnaire towards Others (CQO) were developed and validated (Compassion Questionnaires for Self and Others [CQSO]; Khoury & Vergara, 2024a, 2024b; Khoury et al., 2023). These two questionnaires aimed to measure affective, cognitive, behavioral, and interpersonal skills/abilities that contribute to the cultivation of compassion for self and others, while excluding skills related to mindfulness in order to maintain a distinction between the two concepts and avoid concept contamination (Boateng et al., 2018; Messick, 1995). Each of the two questionnaires (i.e., CQS and CQO) consisted of 18 specific and simple items, each focusing on one single element (emotion, cognition, or behavior) at a time, making them easily understandable by all participants.

Beyond the conceptualization of compassion for self and others, on the operational side, the relationship between self-compassion (SCS) and compassion for others (CS) has been the subject of diverse empirical investigations, yielding mixed results. Some studies have found no significant association between these constructs. For instance, López et al. (2018) reported that compassion for others and self-compassion were not significantly related, with self-compassion being more strongly associated with indicators of affect than compassion for others. Similarly, Mills et al. (2018) found no relationship between self-compassion and compassion for others among palliative care professionals, although significant gender differences were observed, with women reporting lower self-compassion but higher compassion for others than men. A meta-narrative review of the healthcare literature also highlights the absence of evidence linking self-compassion to compassion for others and specifically to the provision of compassionate care, despite this being a postulated outcome of self-compassion interventions (Sinclair et al., 2017).

In contrast, the authors of the compassion scales found small to moderate correlations between self-compassion and compassion for others. For example, the authors of the SOCS found a moderate correlation (*r* = .34; Gu et al., 2020), and a similar moderate association was observed between self-compassion and compassion toward others in the CEAS (Gilbert et al., 2017). However, only a small correlation was found between Neff’s SCS and Pommier’s CS (Pommier et al., 2020). Other studies, not conducted by the original authors of these compassion scales, have reported positive correlations between self-compassion and compassion for others, with the strength of the relationship varying across contexts. For example, Park et al. (2018) found a small positive correlation (*r* = .17) among undergraduate students, whereas Di Fabio and Saklofske (2021) reported a moderate to large positive correlation (*r* = .47) among private-sector workers in Italy. Cultural factors also appear to influence this relationship. In a study conducted in China, Ma and Xiao (2024) found a moderately positive correlation, with self-compassion predicting compassion for others directly and indirectly through psychological resilience and perceived social support. These findings suggest that collectivist and Buddhist cultural frameworks, which emphasize interconnectedness and relational harmony, may promote a closer alignment between self-directed and other-directed compassion (Ma & Xiao, 2024).

Gender differences further complicate the relationship between these constructs. Women consistently report higher levels of compassion for others and lower levels of self-compassion compared to men (López et al., 2018; Mills et al., 2018). Mills et al. (2018) also noted that gender moderated the association between self-compassion and compassion for others, underscoring the importance of demographic factors in shaping these relationships.

On the other hand, most existing compassion scales tend to assess complex abilities that can be considered outcomes or consequences of practicing compassion rather than directly measuring compassion itself. For example, Item 9 in the SCS, “When something upsets me, I try to keep my emotions in balance,” measures emotional regulation. Item 3 of the same scale, “When things are going badly for me, I see the difficulties as part of life that everyone goes through,” reflects a normalization technique widely used in cognitive-behavioral therapy (Cognitive Behavioral Therapy; Beck, 2011). Similarly, Item 1 of the Sussex-Oxford Compassion Scale-Self (SOCSS), “I’m good at recognizing when I’m feeling distressed,” captures emotional awareness, which is closely related to mindfulness practice. Item 9 of the SOCSS, “I connect with my own distress without letting it overwhelm me,” describes a more complex process of acknowledging and regulating emotions.

In summary, these items do not directly describe skills related to cultivating compassion but are primarily consequences of compassion-based practices. In contrast, on the operational level, the new compassion questionnaires differ from existing ones in several key ways: (a) they measure the skills and abilities required to develop compassion rather than the consequences of practicing it; (b) they focus on singular, direct skills instead of complex metacognitive ones, making them easily understandable by participants with different education levels; (c) they include direct behaviors and actions rather than merely an intention or tendency to act; and (d) they include similar yet distinct skills in cultivation compassion for self or others. Overall, the new compassion questionnaires differ from previous ones in both their conceptualization and operationalization of compassion.

Findings from three consecutive studies indicated the merging of the affective and cognitive dimensions, resulting in three distinct dimensions for both the CQS and CQO (Khoury et al., 2023). These dimensions comprise: Thinking/Feeling Compassionately (affective/cognitive factor), Acting or Intending to Act Compassionately (behavioral factor), and Connecting with Others Compassionately (interpersonal factor). Based on the conceptual framework (Khoury, 2019; Khoury & Dionne, 2020), the interpersonal dimension was defined as accepting care or help from others during challenging moments for self-compassion (CQS) and leveraging one’s own suffering to connect with others for the compassion towards others questionnaire (CQO). Validation studies indicated good psychometric properties, including good internal consistency, and provided support for convergent and discriminant evidence (Khoury et al., 2023). In addition, the results suggested that scores on CQS and CQO subscales exhibit moderate associations with mindfulness measures and demonstrate sensitivity to mindfulness training or meditation practice and experience (Khoury et al., 2023).

This operationalization of compassion facilitates the integration of compassion and its measurement into existing cognitive and behavioral interventions, as well as compassion and mindfulness-based programs. In addition, by focusing on the underlying skills/abilities behind compassion, the compassion questionnaires can be complementary to existing compassion measures, which mainly focus on the consequences of compassion. The sensitivity of some of the compassion questionnaire subscales to the meditative experience of participants can also be very useful in studying the influence of meditation experience on the processes involved in compassion training toward self and others.

### Limitation of the Newly Developed Compassion Questionnaires (CQSO)

Despite the many strengths of the newly developed compassion questionnaires (CQSO; Khoury et al., 2023) in conceptualizing and operationalizing compassion for self and others, they also have significant limitations. First, most of the items (except CQS’ item 6 in the Connecting with Others subscale) that were part of the final version of the CQSO were worded in a similar direction (i.e., either positive or negative) for each subscale, which might have limited the full potential of the questionnaires. In fact, the importance of including both positive and negative items in questionnaires has been highlighted in previous research. For instance, numerous studies suggest a two-factor model representing the positive and negative dimensions of the SCS (Coroiu et al., 2018; Costa et al., 2016; López et al., 2015). In addition, research indicates that positive and negative items relate differently to other psychological constructs, with negative items strongly predicting psychopathology, unlike positive ones (Muris & Petrocchi, 2017). Therefore, from an operationalization standpoint, incorporating both positive and negative items is crucial for a comprehensive assessment of compassion.

Second, the number of items per dimension was limited and, in one situation, included only the strict minimum of three items to form a meaningful dimension (i.e., CQS’ Acting Compassionately subscale), which may have reduced the precision of this subscale. In fact, the low number of items, particularly having only three items in one subscale, can pose both theoretical and empirical problems. Theoretically, such a low number of items increases the risk of not fully covering the underlying concept. Empirically, having very few items per subscale (such as three) can lead to poor psychometric properties (El-Den et al., 2020), which was observed in the case of this subscale.

Third, both questionnaires (i.e., CQS and CQO) did not show a global latent variable (or global score), and it was not clear whether the absence of a global latent score was related to the conceptual framework of the questionnaires, lack of compassion exposure among the participants (e.g., practices such as loving-kindness or compassion meditations), limited number of items or lack items coverage of the underlying concepts in some of the dimensions.

Fourth, the CQSO were only validated with women participants, limiting their utility with non-women participants as well as the possibility of comparison based on gender. Fifth, some well-established compassion questionnaires, such as Gilbert’s CEAS (Gilbert et al., 2017), and the recently developed Compassion Scale for others (CS; Pommier et al., 2020), based on Neff’s SCS conceptualization (SCS; Neff, 2003a), were omitted from the validation process of the CQSO to limit the number of administered scales. Including the most common measures of compassion for self and others, such as the CEAS and CS, allows for better and more comprehensive evidence of convergent validity, as well as direct comparison with the new scales.

### The Compassion Questionnaires for Self and Others-Revised

To address the listed limitations of the developed compassion questionnaires (CQSO; Khoury et al., 2023) mentioned above, revised versions of these questionnaires are needed. Such revision aims to achieve the following objectives.

The first objective is to include both positive and negative items when feasible, as described above. Balancing the items of the scales, especially by adding positive items, is based on the theoretical underpinning of compassion, where cultivating love, kindness, and providing care for oneself or others is regarded as an integral part of the related skills. Negative items, such as self-loathing, being critical/judgmental, harsh, or self/other punishing, do not encapsulate the opposite of these positive attributes nor guarantee their measurement. In addition, as mentioned above, positive and negative items relate differently to other measures. Therefore, focusing only on negative items will measure some aspects opposite to compassion but will not fully encapsulate all compassion-based skills. On a statistical level, if items are not well chosen and calibrated, positive items versus negative ones can end up collating together, challenging the proposed structure of the scale. Therefore, from both theoretical and empirical levels, having both positive and negative items allows for a more reliable and valid (at least in terms of face validity) measurement of compassion-related skills and abilities.

The second objective is to enhance the two questionnaires by increasing the number of items and expanding the coverage of the underlying concepts, particularly in dimensions with four items or fewer. This is crucial because a low number of items in a specific dimension, such as only three items, poses a risk to the internal consistency of the scale. Moreover, the limited number of items in both CQS and CQO (each having only 18 items distributed among three dimensions) may restrict the coverage of underlying concepts. This limitation poses a potential threat to the validity and precision of the questionnaires.

Linked with the two aforementioned objectives, the third aim is to retest the presence of a global latent variable (score) for each of the two questionnaires. The presence of a global latent variable or score streamlines the utilization and interpretation of the questionnaires. In addition, this can facilitate the comparison of the questionnaires with other existing ones and enhance their statistical robustness.

The fourth objective is to validate the questionnaires using data collected from both women and non-women participants and to conduct statistical comparisons between the two groups. This is pertinent, as previous studies have indicated potential gender differences in self-compassion (Yarnell et al., 2015, 2019). This objective also involves increasing the number of participants from gender minorities, such as nonbinary and queer.

The fifth objective aims to compare and contrast the new questionnaires with the most frequently cited measures of compassion toward both self and others. This requires the addition of Gilbert’s CEAS (Gilbert et al., 2017), and Pommier’s Compassion Scale for others (CS; Pommier et al., 2020), which were previously omitted. This is important as it can provide better convergence evidence and allow for the comparison of the new questionnaires with all existing compassion scales.

The sixth and final objective is to further delineate the differences and roles of compassion toward self versus toward others, in mental health and global well-being. The study of the links, distinctions, and implications between self-compassion and compassion toward others has been lacking in current literature about compassion (Khoury, 2019; Khoury & Dionne, 2020). This is particularly relevant for the development and validation of new interventions that aim to cultivate compassion toward oneself and others.

Globally, the objectives of the revised questionnaires are to produce better questionnaires on both the theoretical (more coverage of the underlying concepts) and empirical levels (higher psychometrics, validity evidence, and generalizability). In addition, in validating the revised questionnaires, we aimed to incorporate additional methodological approaches such as Machine Learning (ML) to further test the predictive validity of the questionnaires compared to all previously existing compassion measures. In the following sections, we describe the method used to design and develop the revised versions of the compassion questionnaires CQS-R and CQO-R, incorporating the objectives and changes listed above.

## Method

### CQSO-R Design and Development

Based on the six objectives listed above, the existing 18 items of each of the two questionnaires (i.e., CQS and CQO) were expanded to (a) include both positive and negative worded items (when feasible), and (b) increase the number of items to better cover the underlying concepts. The newly added items were edited for language simplicity and clarity by a group of graduate students. This resulted in the inclusion of 59 items in the initially revised version of CQS-R and 64 in the initially revised version of the CQO. Items in both questionnaires were randomly ordered using the “RAND” function in Microsoft Excel before administering the questionnaires to the participants. The instructions related to the administration of the questionnaires remained unchanged from the previous versions. In addition, similarly to the original versions, items were rated on a Likert scale, ranging from 1 (*almost never*) to 5 (*almost always*).

### Participants

A sample of adults from Canada was recruited via paid advertising on social media (e.g., Facebook, LinkedIn, Instagram). Participants were offered $50 gift certificates based on a draw with a rate of winning of 1/10. With the aim of ensuring high external validity and to attain the most conservative sample size, we targeted a sample size of over 1,000 participants (MacCallum et al., 1999). It is fairly accepted that there are no good recommendations for sample size for factor analysis; however, it is accepted that ranges above 500 to 1,000 using item-observation ratios above 1:5 are a conservative and safe approach (Costello & Osborne, 2005; MacCallum et al., 1999; Reio & Shuck, 2015). A total of 1,266 Canadian adult participants were recruited, among them 78.3% were women, 15.6% were men, and 6.1% reported another gender or preferred not to answer. The mean age was 45.2 ± 16.5 years (range from 18 to 87), the mean years of education was 17.6 ± 3.5 years (range from 2 to 35), and the total meditation practice hours were 58.6 ± 117 hr (range from 0 to 599). Detailed descriptions of the sample are available in Table 1.

**Table 1:** Descriptive Results for the Sample Used in the Validation.

Descriptive variable	Overall (*N* = 1,266)
Age	
Mean (SD)	45.2 (16.5)
Median [min, max]	45.0 [18.0, 87.0]
Education in years	
Mean (SD)	17.6 (3.55)
Median [min, max]	18.0 [2.00, 35.0]
Missing	2 (0.2%)
Gender identity	
Woman	991 (78.3%)
Man	197 (15.6%)
Gender variant/non-conforming	42 (3.3%)
Trans	24 (1.9%)
Other	10 (0.8%)
Missing	2 (0.2%)
Sexual orientation	
Heterosexual	902 (71.2%)
Bisexual	148 (11.7%)
Asexual	57 (4.5%)
Gay	34 (2.7%)
Lesbian	34 (2.7%)
Other	60 (4.7%)
Questioning	30 (2.4%)
Missing	1 (0.1%)
Relationship status	
Married	480 (37.9%)
Single	445 (35.2%)
In a relationship	298 (23.5%)
Other	41 (3.2%)
Missing	2 (0.2%)
Living situation	
Family home	577 (45.6%)
Living with significant other	274 (21.6%)
Living alone	257 (20.3%)
Living with roommates	105 (8.3%)
Other	33 (2.6%)
Living without roommates/significant other	15 (1.2%)
Missing	5 (0.4%)
Employment	
Full-time employment	530 (41.9%)
Missing	481 (38.0%)
Part-time employment (30 hr or less per week)	166 (13.1%)
Other	53 (4.2%)
Seasonal/temporary employment	36 (2.8%)
Ethnicity	
White	955 (75.43%)
Indigenous descent	12 (0.95%)
Asian	7 (0.55%)
Latin American	29 (2.29%)
Black	13 (1.03%)
Mixed	92 (7.27%)
Missing	158 (12.48%)
Meditation experience (hr)	
Mean (SD)	58.6 (117)
Median [min, max]	1.90 [0, 599]
Time since started meditation (months)	
Mean (SD)	25.5 (51.9)
Median [min, max]	1.00 [0, 480]
Role of meditation in daily life	
Very important or central in my daily life	39 (3.1%)
Important in my daily life	180 (14.2%)
Little role/importance in my daily life	400 (31.6%)
Somewhat important in my daily life	315 (24.9%)
No role or importance whatsoever	294 (23.2%)
Other	32 (2.5%)
Missing	6 (0.5%)

### Procedure

The study was approved by the Research Ethics Board Office at the first author’s host University. Participants gave informed consent prior to completing the study. The study was conducted entirely online using LimeSurvey and included sociodemographic data with detailed information regarding meditation practice or mindfulness training/experience, along with the measures under validation (i.e., CQSO-R) and a list of other measures for assessing external validity. The order of the questionnaires was not randomized. The newly developed questionnaires were administered first, followed by other compassion and mindfulness questionnaires, and finally, the remaining questionnaires (a comprehensive list of all questionnaires is provided in the following section). Due to the extensive number of questionnaires, participants were given the option to complete all or just a subset. Consequently, our analyses were based solely on the complete responses (details in Table 2).

**Table 2:** Internal Consistencies, Calculated via Cronbach’s Alpha, of Total and Subscale Scores of Instruments Used for Convergent, Discriminant, and Exploratory Evidence.

Description	Number of items	Reliability		Missing (*N* = 1,266)
		Alpha (α)	Omega (ω)	
Self-compassion scale				
Total	26	.96	.96	470 (37.1%)
Self-kindness	5	.89	.89	457 (36.1%)
Self-judgment	5	.88	.88	448 (35.4%)
Common humanity	4	.84	.84	449 (35.5%)
Isolation	4	.83	.83	447 (35.3%)
Mindfulness	4	.83	.83	449 (35.5%)
Overidentified	4	.81	.82	452 (35.7%)
Self-warmth	13	.92	.94	462 (36.5%)
Self-coldness	13	.93	.94	455 (35.9%)
Compassion scale (for others)				
Total	16	.89	.90	380 (30.0%)
Kindness	4	.83	.84	374 (29.5%)
Common humanity	4	.65	.66	373 (29.5%)
Mindfulness	4	.80	.81	373 (29.5%)
Indifference	4	.75	.75	375 (29.6%)
Sussex-Oxford compassion for the self scale				
Total	20	.94	.94	489 (38.6%)
Recognizing suffering	4	.86	.86	479 (37.8%)
Understanding suffering	4	.89	.89	479 (37.8%)
Feeling suffering	4	.87	.87	478 (37.8%)
Tolerating feelings	4	.85	.85	478 (37.8%)
Acting to alleviate	4	.90	.90	477 (37.7%)
Sussex-Oxford compassion for others scale				
Total	20	.94	.94	520 (41.1%)
Recognizing suffering	4	.89	.89	504 (39.8%)
Understanding suffering	4	.91	.91	503 (39.7%)
Feeling suffering	4	.82	.82	503 (39.7%)
Tolerating feelings	4	.77	.78	503 (39.7%)
Acting to alleviate	4	.89	.90	505 (39.9%)
The compassionate engagement and action scales				
Compassion for self	10	.90	.91	559 (44.2%)
Engagement	6	.80	.81	557 (44.0%)
Actions	4	.91	.91	554 (43.8%)
Compassion for others	10	.92	.92	572 (45.2%)
Engagement	6	.84	.85	571 (45.1%)
Actions	4	.89	.89	569 (44.9%)
Compassion from others	10	.95	.95	589 (46.5%)
Engagement	6	.92	.92	589 (46.5%)
Actions	4	.93	.93	584 (46.1%)
Forms of self-criticizing/attacking and self-reassurance scale				
Inadequate self	9	.92	.92	535 (42.3%)
Reassured self	8	.92	.92	542 (42.8%)
Hated self	5	.88	.88	539 (42.6%)
Active-empathic listening scale				
Total	11	.90	.90	617 (48.7%)
Sensing	4	.86	.87	611 (48.3%)
Processing	3	.69	.70	614 (48.5%)
Responding	4	.83	.83	612 (48.3%)
Toronto empathy questionnaire				
Total	16	.90	.91	619 (48.9%)
Social connectedness scale-revised				
Total	20	.94	.94	465 (36.7%)
Social safeness and pleasure scale				
Total	11	.96	.96	593 (46.8%)
Five-factor mindfulness questionnaire				
Total	39	.85	.85	402 (31.8%)
Observe	8	.66	.67	396 (31.3%)
Describe	8	.86	.86	397 (31.4%)
Act with awareness	8	.76	.78	396 (31.3%)
Non-judging	8	.83	.83	394 (31.1%)
Non-reactivity	7	.76	.76	396 (31.3%)
Mindfulness attention and awareness scale				
Total	15	.91	.91	428 (33.8%)
Interpersonal mindfulness scale				
Total	27	.93	.93	533 (42.1%)
Non-reactivity	6	.83	.83	517 (40.8%)
Nonjudgmental acceptance	4	.74	.74	515 (40.7%)
Awareness	10	.88	.88	523 (41.3%)
Presence	7	.89	.89	519 (41.0%)
Perceived stress scale				
Total	10	.92	.92	605 (47.8%)
Depression anxiety stress scales				
Depression	14	.92	.92	607 (47.9%)
Anxiety	14	.83	.83	608 (48.0%)
Stress	14	.86	.86	612 (48.3%)
Positive and negative affect scale				
Positive	10	.90	.90	616 (48.7%)
Negative	10	.91	.91	616 (48.7%)
Emotion-regulation skills questionnaire				
Total	27	.96	.96	638 (50.4%)
Attention toward feelings	3	.79	.79	612 (48.3%)
Body perception of feelings	3	.76	.76	611 (48.3%)
Clarity of feelings	3	.88	.88	612 (48.3%)
Understanding of feelings	3	.84	.85	608 (48.0%)
Acceptance of feelings	3	.82	.83	610 (48.2%)
Resilience: tolerate and endure feelings	3	.85	.85	609 (48.1%)
Readiness to confront undesired emotions	3	.76	.77	612 (48.3%)
Self-support	3	.86	.86	611 (48.3%)
Modification	3	.87	.87	608 (48.0%)
Satisfaction with life scale				
Total	5	.90	.90	616 (48.7%)
Ryff’s scales of psychological well-being				
Autonomy	7	.71	.72	621 (49.1%)
Environmental mastery	7	.80	.81	624 (49.3%)
Personal growth	7	.67	.69	618 (48.8%)
Positive relations with others	7	.76	.78	621 (49.1%)
Purpose in life	7	.79	.80	622 (49.1%)
Self-acceptance	7	.74	.75	621 (49.1%)

### Measures

A total of 21 instruments were administered for convergent, discriminant, and exploratory purposes. The selection of the scales was guided by their theoretical value, their psychometrics, and their use in the validation of previous compassion measures. For convergent evidence, we used other compassion and empathy-related scales including, the SCS (26 items; Neff, 2003a), the Compassion Scale (CS; Pommier et al., 2020), Sussex-Oxford Compassion for Others Scale (SOCSO, 20 items; Gu et al., 2020), Sussex-Oxford Compassion for the Self Scale (SOCSS, 20 items; Gu et al., 2020), Gilbert’s Compassionate Engagement and Action Scales, which include a total of three scales, Compassion for Self, Compassion for Others, and Compassion from Others (CEAS; Gilbert et al., 2017), Self-Criticizing/Attacking & Self-Reassuring Scale (FSCRS, 22 items; Baião et al., 2015), Active-Empathic Listening Scale (AELS, 11 items; Bodie, 2011), and Toronto Empathy Questionnaire (TEQ, 16 items; Spreng et al., 2009). In the same line of convergent evidence, we also used social-related scales as compassion toward others is likely to be related to social connectedness (e.g., Pommier et al., 2020) as measured by Social Connectedness Scale-Revised (SCoS-R, 20 items; Lee & Robbins, 1995), and self-compassion as well as the ability to accept compassion from others is likely to be related to social safeness (e.g., Kelly & Dupasquier, 2016) as measured by Social Safeness and Pleasure Scale (SSS, 11 items; Gilbert et al., 2009).

For discriminant evidence, as mindfulness has been part of most (if not all) previous conceptualizations and operationalizations of compassion and part of most compassion-based training programs, and to explore the relationship between our compassion questionnaires and both intrapersonal and interpersonal mindfulness in comparison with previous compassion measures, we used the following mindfulness scales, the Five Facet Mindfulness Questionnaire (FFMQ, 39 items; Baer et al., 2006), Mindfulness Attention and Awareness Scale (MAAS, 15 items; Brown & Ryan, 2003), and the Interpersonal Mindfulness Scale (IMS, 27 items; Pratscher et al., 2018).

Moreover, we measured the amount of meditation practice as meditation has been a central component in cultivating compassion in both eastern traditions (e.g., Theravada Buddhism) and western interventions (e.g., Mindful Self-Compassion; Neff & Germer, 2013). This amount was computed using four questions: (a) the average number of minutes participants practiced meditation per week (0 if they do not practice meditation), (b) the number of months since participants started practicing meditation (0 for no practice and for less than one month of practice; in the case of the latter, participants were also asked to enter a number between 0 and 0.99 that reflects the percentage of days they practiced during the month), (c) the number of meditation retreats participants attended (0 if they did not attend any meditation retreat), and finally (d) the number of hours participants practiced meditation during the retreats they attended (0, if they did not attend any meditation retreat).

For predictive evidence, self-compassion has been shown to be a significant protector against psychological symptoms, namely stress, anxiety, depression, and negative affect, and to be a predictor of emotional regulation (e.g., Gu et al., 2020; Inwood & Ferrari, 2018; Neff, 2003a), for assessing discriminant validity, we included, Perceived Stress Scale (PSS, 10 items; Cohen et al., 1983), Depression Anxiety Stress Scales (DASS, 42 items; Lovibond & Lovibond, 1995), Positive and Negative Affect Scale (PANAS-GEN, 20 items; Watson et al., 1988), and Emotion-Regulation Skills Questionnaire (ERSQ, 27 items; Berking & Whitley, 2014).

For further predictive and exploratory purposes of the relationship between compassion for self and others and global well-being and in line with previous literature (e.g., Yang et al., 2016; Zessin et al., 2015), the Satisfaction With Life Scale (SWLS, five items; Diener et al., 1985) and Ryff’s Scales of Psychological Well-Being (PWB, 42 items; Ryff, 1989) were used. Internal consistencies calculated using Cronbach’s alpha were computed for all scales and subscales of the instruments used for convergent, discriminant, and exploratory evidence. Overall, coefficient alphas were found to be very good to excellent, indicating that the overall quality of the collected data was very good, see Table 2 for more detailed information.

### Data Analyses

In line with the first and second objectives, to evaluate the structure of the revised questionnaires (CQSO-R), we used a split-half cross-validation procedure using Exploratory Factor Analysis (EFA) and Confirmatory Factor Analysis (CFA). The rationale was to evaluate the theoretically proposed structure using EFA in the first half of the data (train data), generate the adjustments, to then test using CFA in the second half (test data). This allows to check if the same structure would emerge from, allowing us therefore to test the stability of the CQSO-R structure (Bandalos & Finney, 2010; Gerbing & Hamilton, 1996; Orcan, 2018; Schmitt, 2011). For each EFA iteration, given the multidimensional nature of the CQSO-R, we started performing Parallel Analysis and Optimal Coordinates methods to determine the number of factors to extract (Hayton et al., 2004; Raîche et al., 2013). Once this number was determined, we evaluated the multivariate normality of the items using Henze-Zirkler’s test. In the case where multivariate normality was not met, we proceeded using Principal Axis Factorization Extraction instead of Maximum Likelihood (Costello & Osborne, 2005; de Winter & Dodou, 2012). Finally, given the exploratory nature of this analysis and the expectation that at least some of the subscales will be correlated, we used an oblimin rotation. EFA was performed using 1,000 bootstrap iterations to report the mean of the loadings and 95% confidence intervals. This procedure was iteratively implemented to remove crossloaders (loading including confident intervals in more than one factor) and subloaders (presenting all loadings or confident intervals in the range of −0.3, +0.3). In addition, if item was loading in a factor which was not expected by design, it was also removed. This iterative process was also evaluated while jointly taking into consideration theoretical concerns, confidence intervals of the loadings, and crossloading characteristics, exploring solutions without total removal of cross or subloaders, as well as removing some items from the analysis. Finally, two additional criteria were used for item removal. The first concerned the propensity of items to produce Heywood or Ultra-Heywood cases, and the second involved evaluating potential redaction effects driven by the use of negative and positive items. For the first, if Heywood or Ultra-Heywood cases were detected in bootstrap averages or within the 95% CI, we applied item removal based on a criterion where more than of observations scored 5 and 4, or 1 and 2. For the second, we conducted a cluster analysis following the rationale of Ponce et al. (2022, 2023). More details about these two procedures are provided in Supplemental Material Sections 1 and 2.

Once the CQSO-R structure and retained items were defined, we proceeded to evaluate internal consistency using Cronbach’s alpha (Cronbach, 1951). We also included Total Omega following the criticism of many authors about only using Cronbach’s alpha (Dunn et al., 2014; Huysamen, 2006; Peters, 2014; Sijtsma, 2009). Confidence intervals were also provided using 1,000 bootstrap iterations. During internal consistency estimations, we evaluated the effects of the removal of each item on the subscale’s internal consistency. Once the internal consistency analysis was completed, we confirmed our results in the test data using CFA and estimating again internal consistencies, reporting global fits; Root Mean Square Error of Approximation (RMSEA), Standardized Root Mean Square Residual (SRMR), Comparative Fit Index (CFI), and Tucker Lewis Index (TLI) as diagnostics parameters. We also report the EFA structure and internal consistencies of the test data. In line with the third objective of the study, we tested the presence of a global latent variable (score) for each of the two questionnaires. All statistical analyses and data processing were performed with the R project (R Core Team, 2021).

Following factor analyses and in line with the fifth objective of this study, we assessed the convergent and discriminant validity of the CQSO-R according to the theoretically proposed constructs while including the new compassion scales (i.e., CEAS; Gilbert et al., 2017; CS; Pommier et al., 2020).

The general strategy to evaluate convergence was to assess the correlations of CQO-R and CQS-R with SCS, SOCSS, SOCSO, CS, and CEAS. All these instruments evaluate compassion and should correlate with CQS-R and CQO-R. In addition, as previously explained when introducing the construct, compassion is closely related to mindfulness, which is why we expect these constructs to be related to mindfulness. However, self-compassion and compassion for others should exhibit differential patterns, providing evidence of both convergent and discriminant validity. Specifically, self-compassion should correlate with intrapersonal mindfulness (FFMQ, MAAS) but not with IMS. Compassion for others should demonstrate the opposite pattern, associating with interpersonal mindfulness but not with intrapersonal mindfulness.

Furthermore, we expect self-compassion to be related to positive and negative affect (PANNAS) but not to empathy (TEQ) or active listening (AELS). In contrast, compassion for others should exhibit the inverse pattern, being associated with empathy and active listening but not with PANNAS. Finally, to contrast the convergent and discriminant validity of CQO-R and CQS-R, we also evaluated these expected patterns using already validated compassion instruments. Convergent validity was assessed using Pearson correlations of both CQSO-R subscales with the instruments listed above. All correlations were corrected for multiple comparisons using Bonferroni’s correction. Along with the fourth objective, we evaluated gender differences on CQSO-R scores. Contrasts were performed according to the assumptions using either Welch’s Two Sample t-test or the Wilcoxon rank sum test, applying Bonferroni’s correction and reporting Cohen’s d effect size.

From this point on, the following analyses are expected to contribute to deciding whether CQS-R and CQO-R are suitable for use in a specific research context and how they compare to currently available tools. In line with the sixth objective of the study, we explored potential compassion profiles (e.g., high compassion toward self and others, low compassion toward self and others, low compassion toward self and high compassion toward others, low compassion toward self and high compassion toward others) using other compassion scales and those obtained with the CQSO-R. We expected that these profiles would allow us to detect potential compassion profiles and relate them to other variables expected by literature, such as meditative experience, empathy, satisfaction with life, social connectedness, social safeness, as well as symptomatology by means of depression, anxiety, and stress. This analytic strategy has two main goals, the first is to establish if CQSO-R would produce similar profiles compared to other compassion scales and the previous version of CQSO (assessing convergence validity). The second is to test if these profiles will be associated with meditation experience and symptomatology (assessing discriminant validity). As such, this approach allows us to better assess criterion validity. To achieve this objective, we performed a k-means clustering analysis using 25 random start points using the CQSO-R subscales as a first instance and then repeated the process using total scores of SCS, SOCS, CS, and CEAS following the same method used in the validation of the original version of the compassion questionnaires (CQSO; Khoury et al., 2023). To determine the number of clusters, we used the Elbow and the Silhouette method (Saputra et al., 2020), both been reported to be widely used and robust. Clusters were then characterized, and discriminant validity was examined by contrasts using t-test or Mann-Whitney U test according to parametric assumptions (e.g., normality), corrected by Bonferroni’s procedure.

In extending the sixth objective of the study and aligning with the overall objectives to provide better evidence for the revised questionnaires (CQSO-R), we explored predictive evidence using an ML approach. This approach allowed us to evaluate the predictive value of CQSO-R compared to other compassion scales used in this study. Specifically, we utilized Random Forest, which is an extension of Classification and Regression Trees models. Random Forest employs many individual CART models with randomly assigned regressors to predict an outcome variable. These models enable us to obtain variable importance scores, which measure the most influential regressors based on the change in standard error when including that variable in the different CART models comprising the forest (See Supplemental Material Section 3, for a more detailed description of CART models.).

We used conditional random forests (Strobl et al., 2007) and conditional regression trees (Hothorn et al., 2006), both of which use statistical criteria to define each tree split. We categorized the outcome variables into two types: symptoms (i.e., DASS-21 and PANAS) and Well-being (i.e., Ryff and SWLS). All scales were introduced in Z-score so that the change in error prediction (variable importance) could be compared. To improve interpretability, we also ordered predictors based on the median variable importance of each variable’s category (i.e., Symptoms and Well-being). We used the default recommended settings of the party random forest and tree functions (Hothorn et al., 2005, 2006; Strobl et al., 2007, 2008; Zeileis et al., 2008), specifying only an mtry of 5. As predictors, we included all compassion instruments and their subscales.

To further explore the convergent and discriminant value of CQSO-R, we used conditional regression trees with the same dependent variables and only CQSO-R as independent variables. This allowed us to investigate potential interactions between CQSO-R subscales to produce specific scores in symptoms and well-being-related instruments. Conditional regression trees were applied with default parametrization (Hothorn et al., 2005, 2006; Strobl et al., 2007, 2008; Zeileis et al., 2008). See Supplemental Material, Section 3, for a more detailed description of CART models and guidance on interpreting their results.

## Results

### CQSO-R Construct and Reliability Evidence

We developed the second version of CQS-R (59 items) and CQO-R (64 items) with the aim of improving the caveats detected in the previous versions, as stated in the objectives of the current study. Factor analyses were performed with the maximum available data for CQO-R (*n* = 912, missing = 27%) and CQS-R (*n* = 1,067, missing = 15%). In line with the first and second objectives, we started with CQS-R EFA in the training data phase. A total of 20 items were removed during the iterative process performed using EFA. No items were removed due to skewness. Seven items were removed as they formed a fourth factor without the contribution of other items. The remaining items (i.e., 13) were removed due to subloading or crossloading. The solution presented a TLI of 0.91, RMSEA of 0.06, RMSR of 0.03, and a Kaiser-Meyer-Olkin of 0.99. We then evaluated this same structure in the second half using CFA; the solution with 39 items presented a CFI of 0.90, TLI of 0.90, a RMSEA of 0.065, and a SRMR of 0.058. Diagnostics were stable in both halves using EFA and CFA, presenting an adequate fit. As such, CQS-R presented evidence in favor of its structure. We used the same procedure with CQO-R, and 31 items were removed. Specifically, we removed 15 items due to skewness (see Supplemental Material Section 1 for details), 10 items due to crossloading or subloading, 5 items due to incorrect scale loading or loadings with confidence intervals crossing the 0.3 criterion in the training data, and one due to odd behavior during redaction effect analysis (see Supplemental Material Section 2 for details). The solution with 33 items presented a TLI of 0.90, RMSEA of 0.06, RMSR of 0.03, and a KMO of 0.99. We then evaluated this same structure in the second half using CFA, the solution presented a CFI of 0.92, TLI of 0.92, a RMSEA of 0.06, and a SRMR of 0.05. Diagnostics were stable in both halves using EFA and CFA, presenting an adequate fit. For both instruments, EFA loadings 95% confidence intervals were above the 0.3 threshold in the test data. Loadings presented a range between 0.47 and 0.85 for CQS-R and 0.39 to 0.90 for CQO-R in the test data. CQS-R Acting Compassionately was the scale with the lowest internal consistencies (Cronbach’s Alpha: .91 and .93, Total Omega: .92 and .93, first and second half, respectively). For CQO-R Connection with others presented the lowest internal consistencies (Cronbach’s Alpha: .90 and .89, Total Omega: .90 and .89, first and second half, respectively). Factor loadings and internal consistencies are presented in Tables 3 and 4. Despite the usage of positive and negative items, they behaved consistently without presenting evident redaction effects (See Supplemental Material Section 2). A version of the two instruments, along with their factors and scoring, is provided in Appendix A. Altogether results present strong evidence in favor of CQS-R and CQO-R construct validity.

**Table 3:** EFA Results in Training and Test Datasets for CQO-R.

Item	Train dataset			Test dataset		
	Acting or intention to act compassionately	Thinking and feeling compassionately	Connection with others	Acting or intention to act compassionately	Thinking and feeling compassionately	Connection with others
26. When someone struggles, I try to soothe their suffering	0.86 (0.81, 0.92)	−0.06 (−0.13, 0.00)	0.03 (−0.03, 0.09)	0.78 (0.7, 0.86)	0.04 (−0.03, 0.12)	0.05 (0.00, 0.11)
13. During hard times, I take actions to relieve the suffering of others	0.86 (0.79, 0.92)	−0.03 (−0.1, 0.02)	−0.02 (−0.09, 0.04)	0.83 (0.76, 0.9)	0.01 (−0.05, 0.07)	−0.06 (−0.12, 0.00)
43. I try to comfort others when they are suffering	0.84 (0.77, 0.9)	0.01 (−0.05, 0.09)	−0.01 (−0.08, 0.06)	0.86 (0.8, 0.92)	−0.02 (−0.10, 0.04)	0.00 (−0.05, 0.06)
35. When someone is feeling bad, I try to soothe their suffering	0.83 (0.76, 0.9)	0 (−0.06, 0.06)	0.03 (−0.02, 0.10)	0.86 (0.8, 0.92)	−0.02 (−0.09, 0.03)	0.02 (−0.02, 0.07)
27. When someone is feeling bad, I try to soothe them	0.82 (0.75, 0.88)	−0.01 (−0.08, 0.06)	0.04 (−0.03, 0.11)	0.80 (0.74, 0.87)	0.02 (−0.05, 0.09)	0.03 (−0.02, 0.09)
59. I try to help others when they have difficult times	0.80 (0.73, 0.87)	0.03 (−0.04, 0.10)	−0.01 (−0.07, 0.04)	0.81 (0.75, 0.87)	0.04 (−0.02, 0.10)	0.02 (−0.02, 0.06)
9. I try to alleviate the suffering of others when they struggle	0.79 (0.71, 0.88)	0.00 (−0.06, 0.06)	−0.05 (−0.12, 0.02)	0.84 (0.78, 0.9)	−0.02 (−0.09, 0.04)	−0.04 (−0.1, 0.01)
42. During difficult times, I try to console others	0.78 (0.69, 0.86)	0.06 (0, 0.13)	0.00 (−0.07, 0.05)	0.84 (0.77, 0.91)	−0.02 (−0.09, 0.03)	−0.02 (−0.07, 0.03)
34. I help others when they have a tough time	0.77 (0.69, 0.85)	0.05 (−0.01, 0.12)	0.04 (−0.02, 0.11)	0.83 (0.76, 0.9)	0.01 (−0.05, 0.07)	0.01 (−0.04, 0.07)
10. During hard times, I take actions to reduce the pain of others	0.77 (0.69, 0.85)	0.04 (−0.02, 0.11)	0.01 (−0.05, 0.09)	0.81 (0.74, 0.88)	0.01 (−0.05, 0.08)	0 (−0.06, 0.06)
51. I take care of others when they are in need	0.77 (0.69, 0.85)	0.02 (−0.05, 0.1)	0.00 (−0.08, 0.06)	0.76 (0.68, 0.83)	0.07 (0.00, 0.15)	−0.01 (−0.08, 0.04)
22. During hard times, I take actions to relieve my suffering	0.75 (0.66, 0.85)	0.01 (−0.05, 0.09)	0.08 (0.00, 0.15)	0.78 (0.70, 0.87)	0.05 (−0.01, 0.13)	0.04 (−0.01, 0.1)
7. During difficult times, I try to relieve the pain of others	0.71 (0.59, 0.82)	0.01 (−0.07, 0.1)	0.01 (−0.07, 0.11)	0.67 (0.51, 0.83)	0.00 (−0.10, 0.09)	0.11 (0.01, 0.2)
49. I am very critical of other people’s flaws and inadequacies	0.07 (0.00, 0.15)	−0.83 (−0.88, −0.78)	0.02 (−0.05, 0.09)	0.00 (−0.08, 0.09)	−0.79 (−0.85, −0.73)	0.09 (0.01, 0.16)
6. I judge others ruthlessly when they make a mistake	0.04 (−0.05, 0.13)	−0.74 (−0.81, −0.67)	0.04 (−0.03, 0.12)	−0.01 (−0.10, 0.07)	−0.71 (−0.79, −0.63)	0.05 (−0.02, 0.13)
63. I feel irritated by the shortcomings of others	0.04 (−0.04, 0.13)	−0.73 (−0.79, −0.66)	−0.02 (−0.11, 0.06)	0.14 (0.05, 0.24)	−0.77 (−0.84, −0.71)	−0.02 (−0.11, 0.06)
25. I judge others harshly when they make a mistake	−0.06 (−0.16, 0.02)	−0.71 (−0.79, −0.62)	0.00 (−0.08, 0.08)	−0.05 (−0.13, 0.02)	−0.73 (−0.81, −0.64)	0.01 (−0.05, 0.08)
36. I refrain from judging others when they make a mistake	−0.03 (−0.13, 0.07)	0.69 (0.6, 0.77)	0.03 (−0.05, 0.12)	0.03 (−0.05, 0.12)	0.68 (0.6, 0.77)	0.02 (−0.05, 0.10)
45. I am accepting of other people’s mistakes	0.00 (−0.11, 0.09)	0.66 (0.57, 0.75)	0.08 (0, 0.17)	0.02 (−0.07, 0.12)	0.68 (0.60, 0.76)	0.06 (0.00, 0.14)
29. When someone fails, I think they deserve it	−0.1 (−0.2, 0.00)	−0.65 (−0.73, −0.56)	0.00 (−0.08, 0.08)	−0.06 (−0.17, 0.05)	−0.62 (−0.72, −0.53)	−0.05 (−0.13, 0.03)
50. I am very forgiving of other people’s flaws and inadequacies	0.06 (−0.06, 0.18)	0.65 (0.54, 0.76)	0.01 (−0.09, 0.11)	0.05 (−0.04, 0.15)	0.69 (0.59, 0.78)	0.02 (−0.06, 0.11)
21. I feel annoyed at others when they do not understand things quickly	0.11 (0.01, 0.21)	−0.63 (−0.72, −0.53)	−0.04 (−0.15, 0.05)	0.09 (0.00, 0.19)	−0.62 (−0.72, −0.52)	0.00 (−0.09, 0.09)
54. When someone makes a mistake, I feel mad at them	−0.13 (−0.25, −0.02)	−0.61 (−0.7, −0.52)	0.08 (0.00, 0.17)	−0.09 (−0.19, 0.00)	−0.62 (−0.72, −0.53)	0.05 (−0.03, 0.13)
30. When someone has a hard time, I think it is their mistake	−0.11 (−0.24, 0.01)	−0.61 (−0.7, −0.51)	0.01 (−0.09, 0.11)	−0.11 (−0.24, 0.00)	−0.55 (−0.66, −0.43)	−0.05 (−0.15, 0.04)
28. I am nonjudgmental of other people’s failures	0.10 (−0.01, 0.22)	0.60 (0.5, 0.69)	0.04 (−0.04, 0.14)	0.05 (−0.04, 0.14)	0.64 (0.55, 0.73)	0.07 (0.00, 0.15)
20. I can feel positively toward others even after making a mistake	0.19 (0.07, 0.31)	0.51 (0.39, 0.62)	0.03 (−0.05, 0.13)	0.22 (0.11, 0.33)	0.50 (0.39, 0.60)	0.05 (−0.03, 0.13)
57. My struggles do not help me relate to other people’s struggles	−0.07 (−0.2, 0.05)	−0.11(−0.21, −0.02)	−0.54 (−0.67, −0.41)	0.00 (−0.12, 0.10)	−0.17 (−0.26, −0.07)	−0.57 (−0.69, −0.46)
33. My struggles allow me to understand the struggles of others	0.00 (−0.1, 0.08)	0.05 (−0.01, 0.13)	0.76 (0.68, 0.85)	0.07 (−0.01, 0.16)	0.00 (−0.07, 0.07)	0.76 (0.69, 0.83)
4. My suffering helps me connect with other people’s suffering	0.1 (0.00, 0.22)	−0.04 (−0.12, 0.03)	0.68 (0.57, 0.79)	−0.03 (−0.15, 0.07)	−0.02 (−0.12, 0.06)	0.76 (0.67, 0.85)
39. I notice the commonalities between my suffering and the suffering of others	0.03 (−0.06, 0.12)	0.04 (−0.03, 0.12)	0.75 (0.66, 0.83)	0.02 (−0.06, 0.10)	0.05 (−0.02, 0.13)	0.74 (0.66, 0.82)
38. My pain helps me connect with other people’s pain	−0.03 (−0.11, 0.04)	−0.03 (−0.1, 0.02)	0.91 (0.86, 0.96)	0 (−0.07, 0.08)	−0.04 (−0.11, 0.02)	0.86 (0.81, 0.92)
3. Common suffering makes me feel closer to other people during hard times	0.04 (−0.06, 0.15)	−0.02 (−0.12, 0.06)	0.63 (0.53, 0.73)	−0.07 (−0.18, 0.03)	0.05 (−0.03, 0.15)	0.67 (0.59, 0.75)
47. My difficulties make it easier to understand other people’s difficulties	0.01 (−0.06, 0.10)	0.00 (−0.06, 0.06)	0.80 (0.72, 0.87)	0.09 (0.00, 0.19)	−0.05 (−0.13, 0.03)	0.69 (0.60, 0.78)
Cronbach’s alpha	.96 (.96, .97)	.92 (.91, .93)	0.90 (0.88, 0.91)	0.96 (0.95, 0.96)	0.92 (0.91, 0.93)	0.89 (0.87, 0.91)
Total omega	.96 (.95, .96)	.92 (.91, .93)	0.90 (0.88, 0.91)	0.96 (0.95, 0.97)	0.92 (0.91, 0.93)	0.89 (0.87, 0.91)

*Note*. EFA = exploratory factor analysis; CQO-R = compassion questionnaire-others revised.

**Table 4:** EFA in Training and Test Datasets for CQO-R.

Item	Train data set			Test data set		
	Acting or intention to act compassionately	Thinking and feeling compassionately	Connection with others	Acting or intention to act compassionately	Thinking and feeling compassionately	Connection with others
10. During hard times, I take actions to reduce the pain of others	0.77 (0.69, 0.85)	−0.06 (−0.14, 0.0)	0.01 (−0.04, 0.07)	0.76 (0.69, 0.84)	0.01 (−0.05, 0.09)	0.00 (−0.06, 0.07)
13. During hard times, I take actions to relieve the suffering of others	0.82 (0.75, 0.88)	0.00 (−0.08, 0.06)	−0.03 (−0.09, 0.03)	0.83 (0.77, 0.9)	−0.02 (−0.08, 0.03)	−0.01 (−0.07, 0.05)
22. During hard times, I take actions to relieve my suffering	0.80 (0.72, 0.87)	−0.01 (−0.08, 0.05)	0.03 (−0.03, 0.09)	0.81 (0.74, 0.88)	0.00 (−0.06, 0.08)	0.04 (−0.01, 0.1)
26. When someone struggles, I try to soothe their suffering	0.83 (0.76, 0.89)	−0.01 (−0.08, 0.06)	0.00 (−0.07, 0.06)	0.85 (0.78, 0.91)	−0.01 (−0.07, 0.04)	0.02 (−0.02, 0.08)
27. When someone is feeling bad, I try to soothe them	0.82 (0.76, 0.88)	0.04 (−0.02, 0.11)	0.04 (0.0, 0.1)	0.85 (0.79, 0.91)	0.00 (−0.06, 0.06)	0.02 (−0.03, 0.08)
34. I help others when they have a tough time	0.82 (0.75, 0.89)	−0.03 (−0.1, 0.03)	−0.03 (−0.1, 0.02)	0.80 (0.72, 0.88)	0.02 (−0.04, 0.1)	0.04 (−0.01, 0.1)
35. When someone is feeling bad, I try to soothe their suffering	0.85 (0.78, 0.92)	0.01 (−0.04, 0.07)	0.04 (0.0, 0.09)	0.86 (0.81, 0.91)	−0.02 (−0.09, 0.03)	0.00 (−0.05, 0.06)
42. During difficult times, I try to console others	0.79 (0.72, 0.87)	−0.02 (−0.1, 0.04)	0.02 (−0.03, 0.08)	0.88 (0.82, 0.94)	−0.02 (−0.08, 0.03)	−0.01 (−0.06, 0.03)
43. I try to comfort others when they are suffering	0.81 (0.73, 0.88)	0.01 (−0.07, 0.09)	0.03 (−0.03, 0.09)	0.86 (0.8, 0.92)	0.03 (−0.03, 0.09)	−0.01 (−0.06, 0.04)
51. I take care of others when they are in need	0.78 (0.7, 0.85)	−0.03 (−0.11, 0.04)	−0.01 (−0.08, 0.05)	0.79 (0.72, 0.86)	0.05 (−0.01, 0.13)	−0.03 (−0.09, 0.02)
59. I try to help others when they have difficult times	0.82 (0.75, 0.89)	−0.05 (−0.11, 0.01)	−0.03 (−0.08, 0.02)	0.82 (0.76, 0.88)	0.03 (−0.03, 0.1)	0.00 (−0.05, 0.06)
7. During difficult times, I try to relieve the pain of others	0.76 (0.65, 0.87)	0.06 (−0.02, 0.14)	0.01 (−0.07, 0.1)	0.62 (0.48, 0.75)	−0.01 (−0.11, 0.08)	0.09 (0.0, 0.19)
9. I try to alleviate the suffering of others when they struggle	0.78 (0.7, 0.87)	0.01 (−0.06, 0.08)	0.00 (−0.07, 0.08)	0.78 (0.69, 0.86)	0.01 (−0.05, 0.08)	−0.01 (−0.09, 0.05)
20. I can feel positively toward others even after making a mistake	−0.2 (−0.3, −0.11)	0.52 (0.43, 0.61)	−0.02 (−0.1, 0.05)	0.2 (0.08, 0.33)	0.41 (0.29, 0.53)	0.04 (−0.04, 0.13)
21. I feel annoyed at others when they do not understand things quickly	−0.1 (−0.2, −0.01)	−0.64 (−0.73,−0.54)	−0.01 (−0.1, 0.08)	0.02 (−0.08, 0.12)	−0.60 (−0.69,−0.5)	0.02 (−0.07, 0.12)
25. I judge others harshly when they make a mistake	0.06 (−0.01, 0.15)	−0.75 (−0.84,−0.66)	0.00 (−0.07, 0.06)	0.00 (−0.09, 0.09)	−0.71 (−0.79,−0.62)	0.00 (−0.08, 0.07)
28. I am nonjudgmental of other people’s failures	0.00 (−0.1, 0.08)	0.68 (0.59, 0.76)	−0.02 (−0.1, 0.04)	0.05 (−0.06, 0.17)	0.61 (0.52, 0.71)	0.09 (0, 0.18)
29. When someone fails, I think they deserve it	0.06 (−0.03, 0.15)	−0.66 (−0.74,−0.57)	0.00 (−0.07, 0.08)	0.00 (−0.11, 0.11)	−0.64 (−0.73,−0.55)	−0.08 (−0.17, 0)
30. When someone has a hard time, I think it is their mistake	0.13 (0.01, 0.25)	−0.57 (−0.67,−0.46)	0.03 (−0.06, 0.13)	0.08 (−0.03, 0.2)	−0.63 (−0.72,−0.53)	−0.12 (−0.22, −0.03)
36. I refrain from judging others when they make a mistake	−0.03 (−0.12, 0.05)	0.61 (0.51, 0.71)	0.00 (−0.08, 0.09)	−0.05 (−0.15, 0.03)	0.70 (0.62, 0.79)	0.03 (−0.05, 0.12)
45. I am accepting of other people’s mistakes	0.01 (−0.08, 0.11)	0.68 (0.59, 0.77)	−0.01 (−0.09, 0.06)	0.05 (−0.05, 0.17)	0.65 (0.55, 0.75)	0.05 (−0.03, 0.13)
49. I am very critical of other people’s flaws and inadequacies	−0.04 (−0.11, 0.02)	−0.82 (−0.87,−0.76)	−0.03 (−0.1, 0.03)	−0.02 (−0.12, 0.06)	−0.76 (−0.82,−0.69)	0.08 (0, 0.16)
50. I am very forgiving of other people’s flaws and inadequacies	−0.04 (−0.14, 0.04)	0.67 (0.59, 0.76)	−0.04 (−0.12, 0.03)	0.13 (0.01, 0.26)	0.61 (0.49, 0.72)	−0.05 (−0.14, 0.04)
53. The failures of others do not change how I feel toward them	−0.08 (−0.19, 0.01)	0.46 (0.36, 0.57)	0.01 (−0.09, 0.11)	0.15 (0.02, 0.29)	0.41 (0.29, 0.53)	0.00 (−0.11, 0.09)
54. When someone makes a mistake, I feel mad at them	0.09 (0, 0.19)	−0.62 (−0.71,−0.53)	−0.07 (−0.15, 0.01)	−0.04 (−0.16, 0.07)	−0.62 (−0.72,−0.52)	0.05 (−0.04, 0.14)
6. I judge others ruthlessly when they make a mistake	−0.04 (−0.13, 0.04)	−0.72 (−0.8,−0.64)	0.03 (−0.04, 0.1)	−0.03 (−0.12, 0.06)	−0.71 (−0.79,−0.63)	0.06 (−0.01, 0.14)
63. I feel irritated by the shortcomings of others	−0.06 (−0.14, 0.01)	−0.75 (−0.82,−0.68)	0.02 (−0.04, 0.1)	0.06 (−0.03, 0.16)	−0.69 (−0.78,−0.61)	0.00 (−0.09, 0.08)
3. Common suffering makes me feel closer to other people during hard times	0.02 (−0.09, 0.14)	0.02 (−0.06, 0.11)	0.6 (0.51, 0.69)	−0.02 (−0.12, 0.06)	−0.03 (−0.13, 0.06)	0.67 (0.58, 0.76)
33. My struggles allow me to understand the struggles of others	0.08 (0, 0.17)	0.00 (−0.07, 0.07)	0.73 (0.66, 0.8)	0.02 (−0.05, 0.1)	0.07 (0.01, 0.14)	0.77 (0.69, 0.84)
38. My pain helps me connect with other people’s pain	−0.03 (−0.1, 0.04)	0.00 (−0.07, 0.07)	0.85 (0.8, 0.9)	−0.02 (−0.08, 0.03)	−0.01 (−0.07, 0.03)	0.90 (0.85, 0.95)
39. I notice the commonalities between my suffering and the suffering of others	0.00 (−0.08, 0.06)	0.00 (−0.06, 0.06)	0.82 (0.76, 0.87)	0.06 (−0.04, 0.16)	0.09 (0, 0.19)	0.67 (0.58, 0.77)
4. My suffering helps me connect with other people’s suffering	−0.06 (−0.15, 0.03)	0.03 (−0.05, 0.12)	0.73 (0.65, 0.81)	0.06 (−0.03, 0.15)	−0.07 (−0.15, 0.01)	0.76 (0.67, 0.85)
47. My difficulties make it easier to understand other people’s difficulties	0.11 (0.03, 0.19)	0.01 (−0.05, 0.07)	0.75 (0.68, 0.81)	0.02 (−0.06, 0.11)	−0.01 (−0.08, 0.05)	0.75 (0.67, 0.83)
57. My struggles do not help me relate to other people’s struggles	0.01 (−0.08, 0.11)	0.16 (0.07, 0.24)	−0.64 (−0.73,−0.54)	−0.11 (−0.23, 0)	−0.09 (−0.18, 0.00)	−0.51 (−0.63,−0.4)
Cronbach’s alpha	.96 (.95, .96)	.92 (.91, .93)	0.89 (0.88, 0.91)	0.95 (0.95, 0.96)	0.91 (0.90, 0.92)	0.89 (0.87, 0.90)
Total omega	.96 (.95, .96)	.92 (.91, .93)	0.89 (0.88, 0.91)	0.95 (0.95, 0.96)	0.91 (0.90, 0.93)	0.89 (0.87, 0.91)

*Note*. EFA = exploratory factor analysis; CQO-R = compassion questionnaire-others revised.

**Figure 1.**
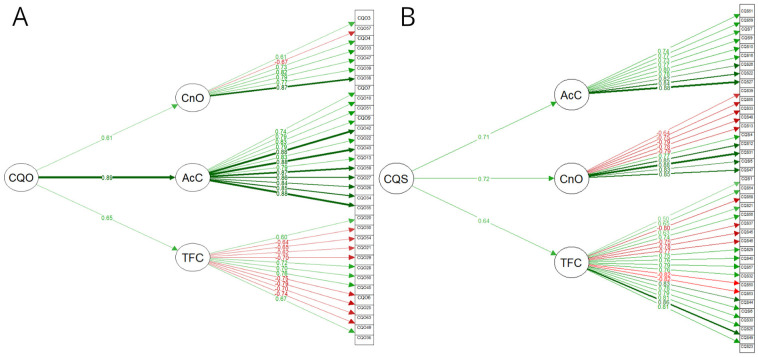
Confirmatory Factor Analysis Results for the (A) CQO-R and (B) CQS-R Scales Solutions. *Note.* Thickness and darkness of the arrows depict the value standardized to all coefficients. Curved arrows between scales denote covariances. Green depicts positive values, while red depicts negative values. CQS-R = compassion questionnaire-self revised; CQO-R = compassion questionnaire-others revised; TFC = thinking and feeling compassionately; AcC = acting compassionately; CnO = connection with others.

In line with the third objective of the study, we explored the plausibility of a global latent variable for CQS-R and CQO-R. When submitting the oblique and the hierarchic versions of CQS to a chi-squared test, no significant difference between both models. Given that the oblique solution did not outperform a hierarchic approach, we concluded that a global score for CQS-R would be informative (Table 5). In the case of CQO-R, the hierarchic solution outperformed the oblique solution, increasing the model’s fit and reducing residuals, confirming the existence of a global latent variable and supporting the use of a global score for CQO-R (Table 5). Final CFA solutions are summarized in Figure 1.

**Table 5:** CFA Diagnostics Using an Oblique and Hierarchical Solution. Difference Stands for the Difference in Fit Index Between Both Solutions.

Scale	Model	TLI	CFI	RMSEA	SRMR	χ^2^	*p*-value
CQO-R	Oblique	0.92	0.92	0.06	0.05	1,284.6	≈1
	Hierarchical	0.92	0.92	0.06	0.05	1,284.6	
	Diff	≈0	≈0	≈0	≈0	≈0	
CQS-R	Oblique	0.902	0.907	0.065	0.058	2,260.6	≈1
	Hierarchical	0.902	0.907	0.065	0.058	2,260.6	
	Diff	≈0	≈0	≈0	≈0	≈0	

*Note*. CFA = confirmatory factor analysis; CQO-R = compassion questionnaire-others revised; CQS-R = compassion questionnaire-self revised; TLI = Tucker Lewis index; CFI = comparative fit index; RMSEA = root mean square error of approximation; SRMR = standardized root mean square residual.

CQS-R total score yielded a Cronbach’s alpha of .91 (95% CI [0.90, 0.92]) and a Total Omega of .93 [0.92, 0.94] for the training dataset, and .91 [0.90, 0.92] and .92 [0.91, 0.93] for the test dataset. For CQO-R total score, we obtained a Cronbach’s alpha of .95 [0.94, 0.96] and a Total Omega of .95 [0.94, 0.95] for the training dataset, and .95 [0.94, 0.96] and .95 [0.94, 0.95] for the test dataset.

### CQSO-R Convergent and Discriminant Evidence

**Figure 2.**
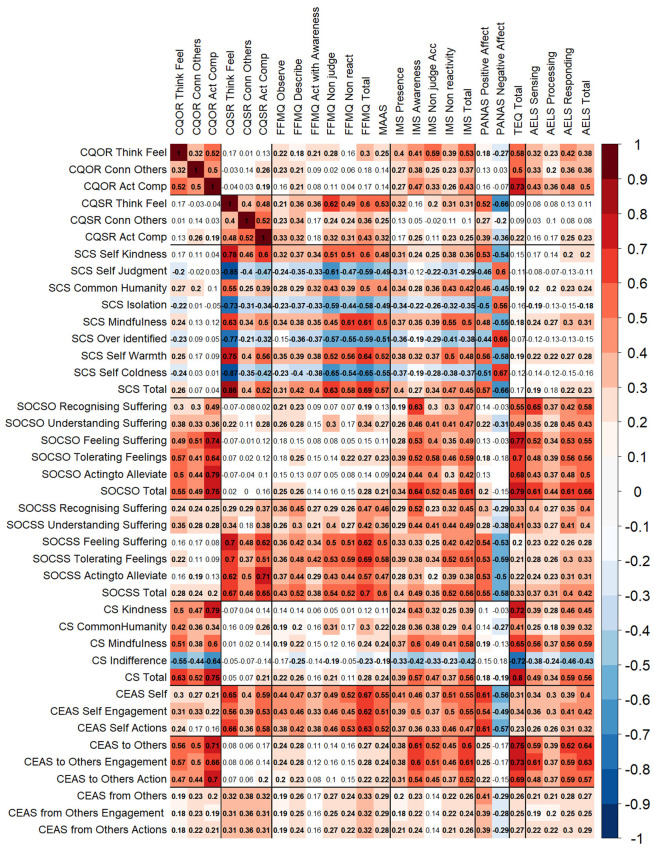
Pearson Correlation Matrix Between the CQS-R and CQO-R Subscales, Other Compassion Instruments (SCS, SOCSO, SOCSS, CS, and CEAS), Intrapersonal Mindfulness (FFMQ and MAAS), IMS, PANAS, Empathy (TEQ), and Active Listening (ALES). *Note.* All correlations *p*-values were corrected by multiple comparisons using Bonferroni’s correction. CQS-R = compassion questionnaire-self revised; CQO-R = compassion questionnaire-others revised; SCS = self-compassion scale; SOCS = Sussex-Oxford compassion scales (S: self, O: others); CS = compassion scale; CEAS = compassion engagement and action scales; FFMQ = five facets mindfulness questionnaire; MAAS = mindfulness attention and awareness scale; IMS = interpersonal mindfulness scale; PANAS = positive affect negative affect schedule; TEQ = Toronto empathy questionnaire; AELS = active-empathic listening scale.

In line with the fourth, fifth, and sixth objectives of the study, we report a correlation matrix of all the compassion instruments used in this study, against intrapersonal mindfulness (FFMQ, MAAS), IMS, PANAS, empathy (TEQ), and AELS. Our results support our initial proposal. CQS is mostly associated with intrapersonal mindfulness and PANAS (Figure 2). Some significant associations appeared where we did not expect them, such as with AELS Responding, AELS Total, and TEQ, particularly associated with acting compassionately. For IMS, some unexpected correlations appeared, such as with CQS-R Thinking and Feeling Compassionately and Acting Compassionately, with many IMS subscales. Nonetheless, these were low correlations, with Pearson’s *r* equal to or below .32.

When compared to other self-compassion instruments (i.e., SCS, SOCSS, and CEAS Self), the discriminant validity of CQS-R was better than the other instruments, which presented similar association strengths regardless of whether the variables were related to intra- or interpersonal mindfulness.

When assessing CQO-R, the results support our initial expectations, where CQO-R is mostly associated with interpersonal mindfulness, empathy, and active listening, but not with PANAS or intrapersonal mindfulness. Nonetheless, some weak correlations were found, mostly between CQO-R Thinking and Feeling Compassionately and intrapersonal mindfulness and PANAS. The association pattern was consistent with other compassion-for-others instruments (see Figure 2). Only minor differences were found among them, with CQO-R presenting a lower correlation strength in active listening compared to other compassion-for-others instruments.

Despite the differences that CQS-R and CQO-R may present with the remaining compassion instruments, they all present moderate to high correlations with the other compassion instruments when directed to the same subject (i.e., self or others), and low association strength when submitted to different subjects. In other words, CQO-R mostly correlated with compassion-for-others instruments but showed no or lower correlations with self-compassion instruments. Inversely, CQS-R presented correlations mostly with self-compassion instruments, while showing no or lower correlations with compassion-for-others instruments. In summary, the correlations provide consistent convergent and discriminant evidence supporting that the CQS-R and CQO-R constructs were properly delimited to measure self- and other compassion.

Some gender differences were previously identified in Neff’s measures of self-compassion (Yarnell et al., 2015, 2019). Additionally, gender has traditionally been associated with empathy, suggesting that women tend to exhibit higher levels of empathy (Christov-Moore et al., 2014). Thus, it is plausible to hypothesize gender differences in compassion toward others. To investigate these hypotheses in line with the fourth objective, we examined gender differences in CQSO-R. Interestingly, the most notable differences were observed in CQO-R, whereas CQS-R did not present any significant difference (see Table 6). CQO-R Thinking and Feeling Compassionately was the only nonsignificant CQO-R subscale, while CQO-R Connection with Others was significant, presenting a medium effect size, and CQO-R Acting or Intention to Act Compassionately showed a high effect size. The obtained results are consistent with those found regarding empathy. A similar pattern can be observed for CS, SCS, and SOCS. CEAS presented the most different behavior, presenting significant differences only in the scores related to others. In general, self-compassion-related scales and subscales present nonsignificant results or lower effect sizes. Despite the conceptual differences among compassion instruments, the results present a consistent pattern supporting convergent evidence in favor of CQSO-R and further delineating the differential role of gender on self-compassion and compassion for others.

**Table 6:** Descriptive Statistics and *t*-Test Results of the Differences in CQO-R and CQS-R Subscales by Gender.

Description	Man (*N* = 197)	Woman (*N* = 991)	*p*-value (Cohen’s *D*)
CQO-R thinking and feeling compassionately			
Mean (SD)	3.72 (0.733)	3.90 (0.618)	.231 (0.26)
Median [min, max]	3.73 [2.08, 5.00]	3.92 [1.69, 5.00]	
Missing	57 (28.9%)	241 (24.3%)	
CQO-R connection with others			
Mean (SD)	3.46 (0.901)	3.76 (0.734)	.007 (0.37)
Median [min, max]	3.43 [1.00, 5.00]	3.71 [1.14, 5.00]	
Missing	56 (28.4%)	239 (24.1%)	
CQO-R acting or intention to act compassionately			
Mean (SD)	3.70 (0.787)	4.13 (0.680)	<.001 (0.58)
Median [min, max]	3.77 [1.92, 5.00]	4.08 [1.69, 5.00]	
Missing	56 (28.4%)	241 (24.3%)	
CQS-R thinking and feeling compassionately			
Mean (SD)	2.95 (0.870)	2.82 (0.855)	.09 (0.14)
Median [min, max]	3.00 [1.00, 4.75]	2.85 [1.00, 4.95]	
Missing	34 (17.3%)	107 (10.8%)	
CQS-R connection with others			
Mean (SD)	3.01 (0.884)	2.85 (0.788)	.70 (0.19)
Median [min, max]	3.00 [1.00, 4.80]	2.90 [1.00, 5.00]	
Missing	34 (17.3%)	100 (10.1%)	
CQS-R acting or intention to act compassionately			
Mean (SD)	3.38 (0.800)	3.50 (0.695)	1 (0.16)
Median [min, max]	3.33 [1.00, 5.00]	3.44 [1.00, 5.00]	
Missing	33 (16.8%)	100 (10.1%)	
CS kindness			
Mean (SD)	3.96 (0.807)	4.36 (0.642)	<.001 (0.54)
Median [min, max]	4.00 [1.50, 5.00]	4.50 [2.00, 5.00]	
Missing	64 (32.5%)	283 (28.6%)	
CS common humanity			
Mean (SD)	4.09 (0.727)	4.29 (0.597)	.16 (0.31)
Median [min, max]	4.25 [2.00, 5.00]	4.50 [1.75, 5.00]	
Missing	63 (32.0%)	283 (28.6%)	
CS mindfulness			
Mean (SD)	4.00 (0.651)	4.27 (0.625)	<.001 (0.42)
Median [min, max]	4.00 [1.75, 5.00]	4.50 [1.50, 5.00]	
Missing	63 (32.0%)	283 (28.6%)	
CS indifference			
Mean (SD)	2.23 (0.955)	1.84 (0.716)	<.001 (0.45)
Median [min, max]	2.25 [1.00, 4.75]	1.75 [1.00, 4.50]	
Missing	63 (32.0%)	285 (28.8%)	
CS total			
Mean (SD)	3.96 (0.646)	4.27 (0.517)	<.001 (0.53)
Median [min, max]	4.06 [2.06, 5.00]	4.38 [2.31, 5.00]	
Missing	64 (32.5%)	289 (29.2%)	
CEAS self			
Mean (SD)	62.5 (16.5)	63.5 (16.2)	1 (0.06)
Median [min, max]	64.0 [20.0, 97.0]	65.0 [16.0, 99.0]	
Missing	89 (45.2%)	431 (43.5%)	
CEAS self engagement			
Mean (SD)	37.0 (9.57)	38.2 (9.39)	1 (0.12)
Median [min, max]	37.0 [11.0, 60.0]	39.0 [10.0, 60.0]	
Missing	88 (44.7%)	430 (43.4%)	
CEAS self actions			
Mean (SD)	25.7 (8.26)	25.4 (7.89)	1 (0.04)
Median [min, max]	26.0 [8.00, 40.0]	26.0 [4.00, 40.0]	
Missing	87 (44.2%)	428 (43.2%)	
CEAS to others			
Mean (SD)	69.0 (15.4)	77.8 (13.1)	<.001 (0.61)
Median [min, max]	69.0 [27.0, 100]	80.0 [29.0, 100]	
Missing	90 (45.7%)	440 (44.4%)	
CEAS to others engagement			
Mean (SD)	40.7 (9.48)	45.6 (8.30)	<.001 (0.55)
Median [min, max]	40.0 [16.0, 60.0]	47.0 [16.0, 60.0]	
Missing	89 (45.2%)	440 (44.4%)	
CEAS to others action			
Mean (SD)	28.3 (6.79)	32.2 (5.50)	<.001 (0.62)
Median [min, max]	30.0 [8.00, 40.0]	33.0 [11.0, 40.0]	
Missing	90 (45.7%)	437 (44.1%)	
CEAS from others			
Mean (SD)	56.6 (17.7)	58.4 (19.6)	1 (0.09)
Median [min, max]	57.0 [12.0, 97.0]	59.0 [10.0, 100]	
Missing	95 (48.2%)	450 (45.4%)	
CEAS from others engagement			
Mean (SD)	33.4 (10.6)	34.2 (11.6)	1 (0.06)
Median [min, max]	34.0 [7.00, 57.0]	34.0 [6.00, 60.0]	
Missing	95 (48.2%)	450 (45.4%)	
CEAS from others actions			
Mean (SD)	23.3 (7.74)	24.2 (8.59)	1 (0.11)
Median [min, max]	23.0 [4.00, 40.0]	25.0 [4.00, 40.0]	
Missing	94 (47.7%)	446 (45.0%)	
SCS Self-kindness			
Mean (SD)	3.22 (0.972)	3.04 (0.927)	.76 (0.18)
Median [min, max]	3.40 [1.00, 5.00]	3.00 [1.00, 5.00]	
Missing	76 (38.6%)	345 (34.8%)	
SCS self-judgment			
Mean (SD)	3.10 (0.960)	3.32 (0.989)	.019 (0.23)
Median [min, max]	3.20 [1.00, 5.00]	3.40 [1.00, 5.00]	
Missing	76 (38.6%)	336 (33.9%)	
SCS common humanity			
Mean (SD)	3.19 (0.926)	3.06 (0.943)	1 (0.13)
Median [min, max]	3.25 [1.00, 5.00]	3.00 [1.00, 5.00]	
Missing	76 (38.6%)	336 (33.9%)	
SCS isolation			
Mean (SD)	3.06 (1.01)	3.27 (1.01)	1 (0.21)
Median [min, max]	3.00 [1.00, 5.00]	3.25 [1.00, 5.00]	
Missing	75 (38.1%)	338 (34.1%)	
SCS mindfulness			
Mean (SD)	3.39 (0.862)	3.20 (0.851)	1 (0.22)
Median [min, max]	3.50 [1.75, 5.00]	3.25 [1.00, 5.00]	
Missing	76 (38.6%)	337 (34.0%)	
SCS over identified			
Mean (SD)	2.88 (0.974)	3.19 (0.961)	.04 (0.32)
Median [min, max]	3.00 [1.00, 5.00]	3.25 [1.00, 5.00]	
Missing	76 (38.6%)	339 (34.2%)	
SCS self-warmth			
Mean (SD)	3.27 (0.817)	3.09 (0.813)	.65 (0.21)
Median [min, max]	3.31 [1.38, 4.92]	3.08 [1.08, 5.00]	
Missing	78 (39.6%)	348 (35.1%)	
SCS self-coldness			
Mean (SD)	3.02 (0.902)	3.27 (0.896)	.15 (0.27)
Median [min, max]	3.12 [1.23, 4.92]	3.31 [1.00, 5.00]	
Missing	77 (39.1%)	341 (34.4%)	
SCS total			
Mean (SD)	3.13 (0.798)	2.91 (0.803)	.14 (0.27)
Median [min, max]	3.12 [1.38, 4.85]	2.88 [1.08, 4.96]	
Missing	80 (40.6%)	353 (35.6%)	
SOCSO recognizing suffering			
Mean (SD)	13.9 (2.90)	15.5 (2.66)	<.001 (0.59)
Median [min, max]	14.0 [6.00, 20.0]	16.0 [8.00, 20.0]	
Missing	78 (39.6%)	389 (39.3%)	
SOCSO understanding suffering			
Mean (SD)	16.4 (3.28)	17.5 (2.72)	.02 (0.36)
Median [min, max]	17.0 [8.00, 20.0]	18.0 [7.00, 20.0]	
Missing	78 (39.6%)	388 (39.2%)	
SOCSO feeling suffering			
Mean (SD)	15.0 (2.92)	16.6 (2.44)	<.001 (0.59)
Median [min, max]	15.0 [7.00, 20.0]	17.0 [7.00, 20.0]	
Missing	78 (39.6%)	388 (39.2%)	
SOCSO tolerating feelings			
Mean (SD)	14.7 (2.92)	15.7 (2.43)	.003 (0.38)
Median [min, max]	15.0 [8.00, 20.0]	16.0 [6.00, 20.0]	
Missing	80 (40.6%)	386 (39.0%)	
SOCSO acting to alleviate			
Mean (SD)	15.0 (2.94)	16.4 (2.70)	<.001 (0.52)
Median [min, max]	15.0 [8.00, 20.0]	16.0 [8.00, 20.0]	
Missing	79 (40.1%)	389 (39.3%)	
SOCSO total			
Mean (SD)	74.8 (12.8)	81.8 (10.4)	<.001 (0.59)
Median [min, max]	76.0 [40.0, 100]	82.5 [50.0, 100]	
Missing	80 (40.6%)	403 (40.7%)	
SOCSS recognizing suffering			
Mean (SD)	14.2 (3.14)	14.8 (2.89)	1 (0.18)
Median [min, max]	14.0 [7.00, 20.0]	15.0 [4.00, 20.0]	
Missing	76 (38.6%)	367 (37.0%)	
SOCSS understanding suffering			
Mean (SD)	15.9 (3.22)	16.8 (2.99)	.16 (0.28)
Median [min, max]	16.0 [9.00, 20.0]	17.0 [6.00, 20.0]	
Missing	78 (39.6%)	365 (36.8%)	
SOCSS feeling suffering			
Mean (SD)	13.0 (3.49)	12.5 (3.34)	1 (0.13)
Median [min, max]	13.0 [5.00, 20.0]	12.0 [4.00, 20.0]	
Missing	76 (38.6%)	366 (36.9%)	
SOCSS tolerating feelings			
Mean (SD)	12.8 (3.52)	12.0 (3.29)	.53 (0.24)
Median [min, max]	13.0 [5.00, 20.0]	12.0 [4.00, 20.0]	
Missing	78 (39.6%)	364 (36.7%)	
SOCSS acting to alleviate			
Mean (SD)	13.8 (3.63)	13.4 (3.29)	1 (0.1)
Median [min, max]	14.0 [5.00, 20.0]	13.0 [4.00, 20.0]	
Missing	77 (39.1%)	364 (36.7%)	

*Note*. CQS-R = compassion questionnaire-self revised; CQO-R = compassion questionnaire-others revised; CS = compassion scale; CEAS = compassion engagement and action scales; SCS = self-compassion scale; SOCS = Sussex-Oxford compassion scales (S: self, O: others).

**Figure 3.**
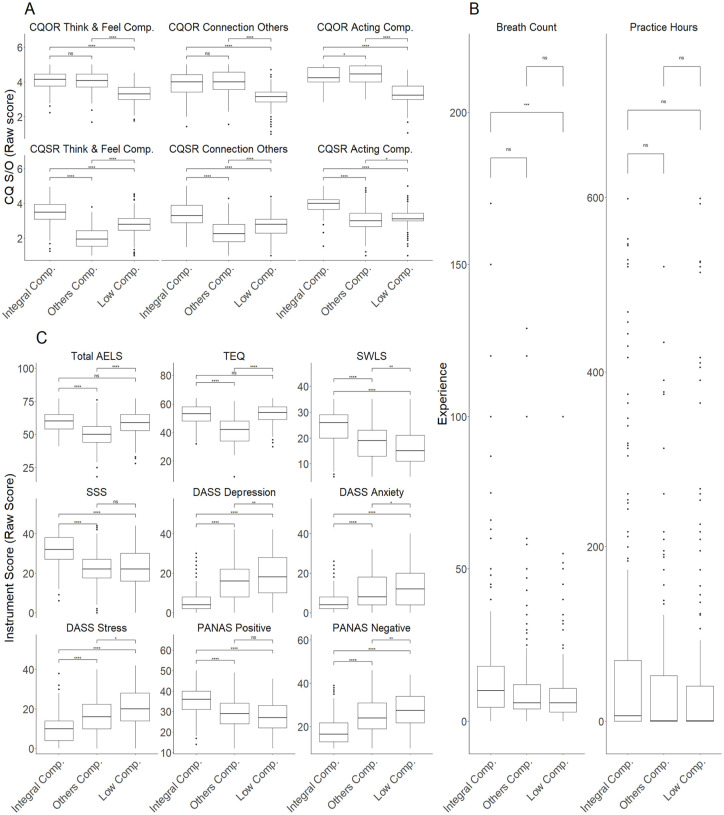
Characterization of CQSO-R-Based Profiles by Means of (A) CQSO-R Subscales Scores, (B) Meditation Experience, and (C) Other Relevant Variables Such as Empathy, Social Connectedness, Satisfaction with Life, and Symptomatology. *Note*. Differences were evaluated using *t*-test or Mann-Whitney U test according to assumption evaluations. CQSO-R = compassion questionnaires for self and others-revised; AELS = active-empathic listening scale; TEQ = Toronto empathy questionnaire; SWLS = satisfaction with life scale; SSS = social safeness scale; DASS = depression anxiety stress scales; PANAS = positive affect negative affect schedule. Nonsignificant results are presented as “ns.” *p < .05. **p < .01. ***p <* .*001.

To further explore CQSO-R convergent and discriminant evidence and the interactions between CQS-R and CQO-R, in line with the fifth and sixth objectives, we developed compassion profiles using a clustering procedure based only on the CQSO-R subscales. Both methods, the Elbow and the Silhouette methods, suggested a three-cluster solution that produced three profiles: (a) Integral Compassion (*n* = 367), which is characterized by high scores in all CQSO-R scales, (b) Others Compassion (*n* = 262), which is characterized by high compassion to others but relatively low compassion toward self, and (c) Low Compassion (*n* = 269), which is characterized by having the low scores in all CQSO-R scales (see Figure 3A). Only integral compassion presented a higher experience by means of Breath count, but it did not significantly differ from the Low compassion groups (Figure 3B). Therefore, previous mindfulness experience does not seem to be a relevant element to distinguish these groups. When examining the AELS, both groups with high compassion for others were significantly higher than the low compassion group (Figure 3C). This same effect was observed for empathy (TEQ). Interestingly enough, for SWLS, social support, and symptomatology (DASS, PANAS), we observed the same pattern, where integral compassion presents the highest benefits, while others compassion and Low compassion groups presented fairly similar scores.

**Figure 4.**
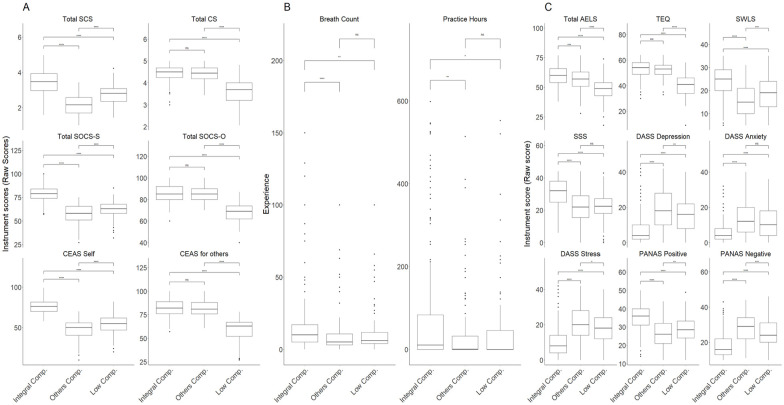
Characterization of SCS, CS, and SOCSS/O, CEAS-Based Profiles by Means of (A) Compassion for Self and Others Scales Scores, (B) Mediation Experience, and (C) Other Relevant Variables Such as Empathy, Social Connectedness, Satisfaction with Life, and Symptomatology. *Note*. Differences were evaluated using *t*-test or Mann-Whitney U test according to assumption evaluations. SCS = self-compassion scale; CS = compassion scale; SOCS = Sussex-Oxford compassion scales (S: self, O: others); CEAS = compassion engagement and action scales; AELS = active-empathic listening scale; TEQ = Toronto empathy questionnaire; SWLS = satisfaction with life scale; SSS = social safeness scale; DASS = depression anxiety stress scales; PANAS = positive affect negative affect schedule. Nonsignificant results are presented as “ns.” *p < .05. **p < .01. ***p < .001.

When replicating the cluster analysis with SOCS-SO, SCS, CS, and CEAS, we found the same three profiles as described above (Figure 4A). However, in this case, the profiles were more sensitive to meditation experience, presenting significant differences from the integral compassion group (Figure 4B). This difference was expected as CQSO-R is better delimited from mindfulness, while the remaining instruments are more related to dispositional mindfulness construct, including a mindfulness dimension in their conceptualization and operationalization, and therefore proxies of their training. For the empathy, satisfaction with life, social connectedness, and symptomatology, we observed the same pattern, where empathy was similar or equivalent in the Integral compassion and other compassion groups, while Low compassion displayed lower scores (Figure 4C). Symptomatology was fairly similar for other compassion and Low compassion groups, presenting the highest benefits in the Integral compassion group. These findings provide robust convergent evidence, indicating that virtually identical profiles emerged whether using only CQSO-R or incorporating all other compassion subscales.

**Figure 5.**
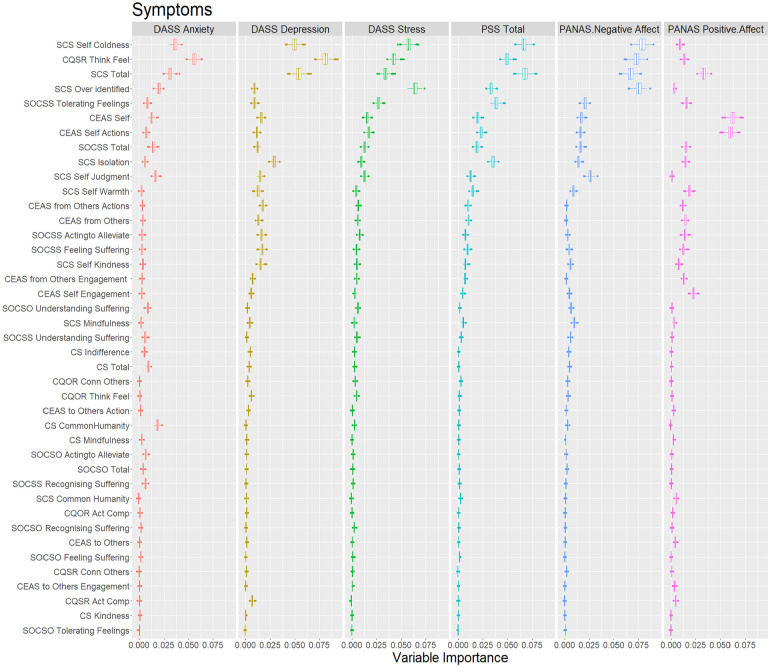
Variable Importance of Each Compassion for Self or Other Measure Subscales When Predicting Symptoms of Stress, Anxiety, Depression, and Positive/Negative Affect. *Note*. Variable importance was ordered using the median value of variable importance across all symptoms’ measures.

When assessing variable importance using conditional random forest, we found that the best predictors for symptomatology were SCS Self-Coldness, CQS-R Thinking/Feeling Compassionately, SCS total score, and SCS over Identified (Figure 5). These variables were consistently good predictors of DASS-21, PSS, and PANAS Negative Affect. PANAS Positive Affect was mostly predicted by CEAS Self and CEAS Self Actions. As expected, symptoms were mostly predicted by self-compassion.

**Figure 6.**
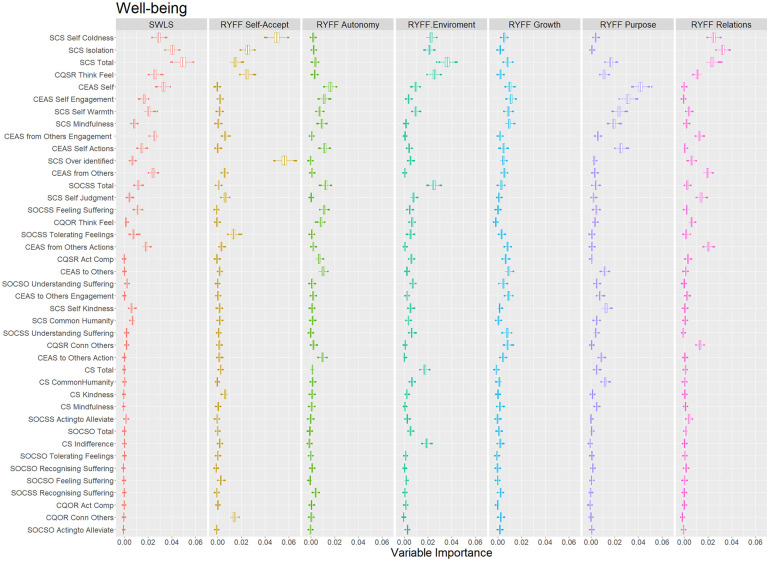
Variable Importance of Each Compassion for Self or Other Measure Subscales When Predicting Well-Being Measures (Such as Satisfaction with Life and Ryff’s Subscales). *Note*. Variable importance was ordered using the median value of variable importance across all well-being measures.

For well-being, we found a less consistent pattern, in which results changed according to the specific well-being scale or subscale evaluated (Figure 6). Nonetheless, there is consistency that the most relevant predictors are associated with self-compassion, including subscales from SCS, CQS-R, and CEAS.

**Figure 7.**
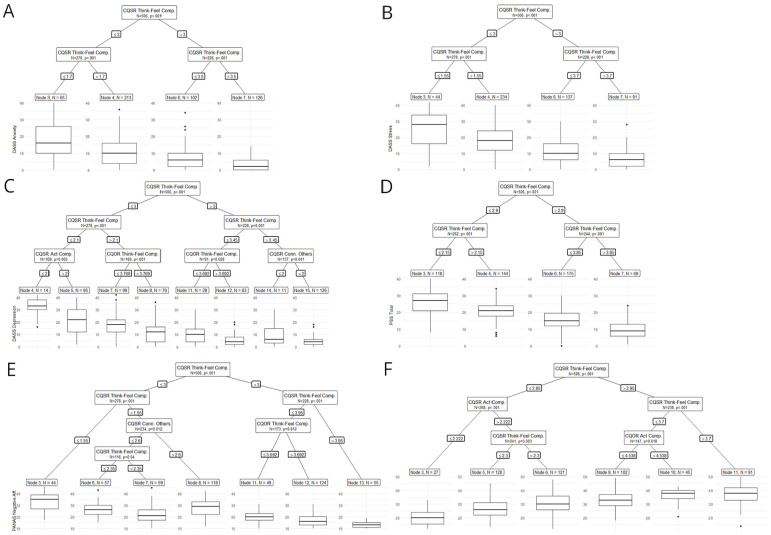
Conditional Regression of Trees Results. Sample Size is Presented (*N*) Along with the *p*-Value for Each Split. All Six Variables Obtained from Figure 5 Were Tested. Only Significant Splits are Reported. Trees are Arranged According to the Variable They Predict: (A) DASS Anxiety, (B) DASS Stress, (C) DASS Depression, (D) PSS, (E) SCoS-R, (E) PANAS Negative Affect, and (F) PANAS Positive Affect. *Note*. DASS = depression anxiety stress scales; PSS = perceived stress scale; SCoS-R = social connectedness scale-revised; PANAS = positive affect negative affect schedule.

**Figure 8.**
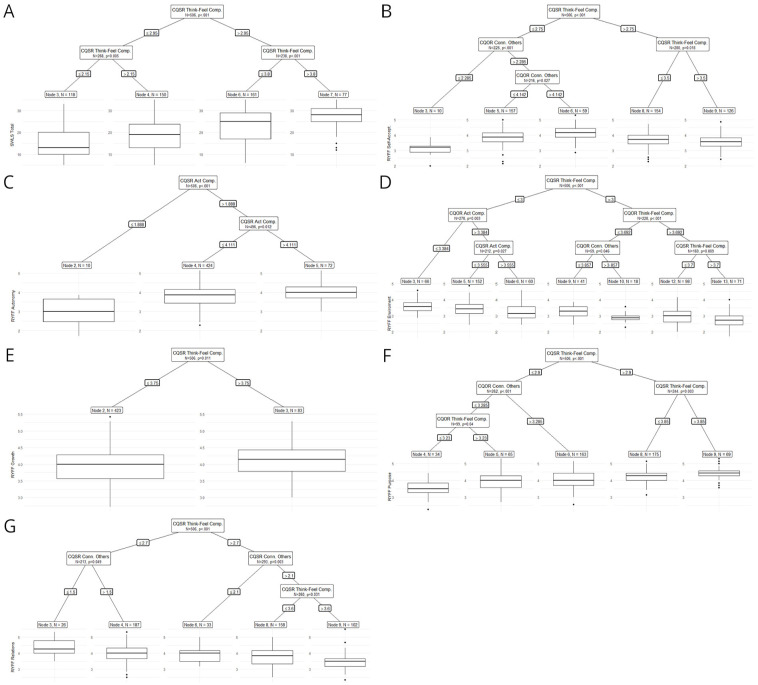
Conditional Regression of Trees Results. Sample Size is Presented (*N*) Along with the *p*-Value for Each Split. All Seven Variables Obtained from Figure 6 were Tested. Only Significant Splits are Reported. Trees are Arranged According to the Variable They Predict: (A) SWLS, (B) Ryff Self-Accept, (C) Ryff Autonomy, (D) Ryff Environmental Mastery, (E) Ryff Growth, (F) Ryff Purpose in Life, (G) Ryff Relations with Others. *Note*. SWLS = satisfaction with life scale.

When evaluating the interactions associated with symptoms using CQOS-R, we found a central role of CQS-R Thinking/Feeling Compassionately, being the root in all the trees evaluated and appearing repeatedly in many splits (Figure 7). In some cases, such as PSS, DASS anxiety, and DASS Stress, CQS-R Thinking/Feeling Compassionately alone predicts those scores. In cases such as DASS Depression and PANAS, scores are predicted by complex interactions between CQS-R and CQO-R subscales. A similar pattern can be observed when evaluating well-being (Figure 8), with the only exception of Ryff autonomy, which was predicted by CQS-R Act Compassionately. In general, both conditional random forest and conditional regression tree results highlight the role of both self-compassion and compassion for others in mental health symptoms and well-being, emphasizing the need of assessing both.

## Discussion

The central aim of the current study was to develop and validate new, revised versions of the compassion questionnaire that address the limitations found in the original versions of the questionnaires. This included a set of six objectives, as outlined at the beginning of this paper.

The first two objectives were to incorporate both positive and negative items whenever possible (Objective One) and to extend the number of items to enhance their coverage of the underlying concepts of the questionnaires (Objective Two). These objectives were largely achieved in the final version of the revised questionnaires. The changes led to better coverage and clarity of the underlying concepts and the inclusion of both positive and negative items when applicable, without redaction effects detected derived from those additions. These modifications were made strategically to minimize unnecessary changes to items that had already demonstrated high performance and psychometrics in the original version. Consequently, the final revised version of the Self-Compassion Questionnaire (CQS-R) now comprises 39 items, and the Compassion towards Others Questionnaire (CQO-R) includes 33 items. The significant increase in the number of items is primarily due to the inclusion of both positively and negatively worded items, as well as the addition of items to subscales that had a very low number of items (four or fewer) in the original version. This expansion ensures better coverage of the underlying affective, cognitive, behavioral, and interpersonal concepts in the revised versions. For detailed information regarding the changes to the original version of the questionnaires, please refer to Appendix B.

With the increase in the item count and the length of certain items, the complexity of both the items and the revised questionnaires also increased. For instance, in the CQS-R, the reading level rose from 3.5 (for the CQS) to 6.3, as per the Microsoft Word Statistics Tool. However, despite this increase, the CQS-R retained a simpler comprehension and response format for participants compared to the SCS, which has a reading level of 7.5. In addition, as shown in the results, the internal structure and consistency of both the CQS-R and CQO-R were superior to the original versions, with all subscales exhibiting higher alpha and omega values. For example, the internal consistency of the Acting Compassionately subscale increased from an average of 0.78 in the original version of the CQS to an average of 0.92 in the CQS-R (CQSO; Khoury et al., 2023). Similarly, the internal consistency of Acting Compassionately in the CQO improved from an average of 0.86 in the original version to an average of 0.96 in the CQO-R (CQSO; Khoury et al., 2023). These results, among others, confirm the increased robustness of the revised versions of the questionnaires.

In line with the third objective, the results show that both revised questionnaires (i.e., CQS-R and CQO-R) present a global latent variable. The absence of such a latent variable was a limitation and a point of criticism of the original compassion questionnaires (CQSO; Khoury et al., 2023). The presence of a global latent variable (and the computation of a global score) for both CQS-R and CQO-R facilitates their use, and comparison with other existing CSs, and enhances their statistical robustness. Despite the utility of the global score for both self-compassion (CQS-R) and compassion for others (CQO-R), it is highly recommended to utilize the scores of each of the three dimensions of the scales (Thinking/Feeling, Acting, and Connecting) in addition to the total score. This is because the individual dimensional scores offer greater detail and granularity, allowing for a more nuanced measurement of the specific skills associated with each facet of self-compassion and compassion for others. These sets of skills, although related, differ in terms of the strategies and practices needed to cultivate them. For instance, nurturing compassionate thoughts and feelings may be better achieved through meditative practices such as Loving-Kindness Meditation, while fostering compassionate actions may require direct behavioral changes. Establishing connections with others may involve various personal and interpersonal elements, necessitating the use of multiple strategies and practices to cultivate them effectively.

The fourth objective aimed to extend the validation of the questionnaires to include non-women participants and to conduct gender-based comparisons. While this objective was mostly achieved, the disparity in participant numbers persisted, with significantly more women than men. This skew was primarily due to greater interest among women in the research topic (i.e., compassion) and the recruitment method via social media. Despite this gender disparity, comparison analyses were successfully conducted. Results revealed higher levels of compassion toward others among women compared to men, consistent with previous research on related concepts such as empathy (Christov-Moore et al., 2014). This is also consistent with the results from previous compassion-for-others scales (CS, SOCSO, and CEAS for others), where the majority of scales and subscales showed higher values for women compared to men, with overall medium to large effect sizes. Although the differences in mean values for the CQO-R Thinking and Feeling Compassionately subscale based on gender did not reach statistical significance, women still had higher mean values (3.90) compared to men (3.71). No significant differences were observed among all the self-compassion subscales. These results on the role of gender in self-compassion are consistent with previous findings based on Neff’s SCS (Yarnell et al., 2015, 2019), which suggested slightly higher levels of self-compassion among men in comparison with women, with a small effect size (*d* = 0.18). In addition, in our sample, other SCS (SCS, CEAS, and SOCSS) did not show any significant overall differences based on gender, except for two negative subscales of Neff’s SCS (self-judgment and over-identification), where men scored slightly lower than women, with small effect sizes. However, these differences did not extend to the total SCS scores, where no significant gender differences were observed. This suggests that while men may report marginally higher self-compassion overall, the differences are subtle and not consistently present across all subscales.

According to the fifth objective, the validation study included Gilbert’s CEAS (Gilbert et al., 2017), and Pommier’s Compassion Scale for others (CS; Pommier et al., 2020), which were omitted in the validation of the original compassion questionnaires (CQSO; Khoury et al., 2023) to limit the number of administered scales. The incorporation of the CEAS and CS bolstered the convergent evidence in our study. Specifically, both CEAS Self and CEAS-O, as well as CS, exhibited high correlations with CQS-R and CQO-R, as outlined in the results section. Moreover, when examining the compassion profiles, these additional questionnaires (i.e., CEAS and CS) demonstrated comparable behavior to other compassion measures, including the revised questionnaires. In this context, CQS-R stands out from the other self-compassion instruments by exhibiting better delimited behavior, particularly in terms of discriminant validity, supported by both convergent and discriminant evidence. Furthermore, results using conditional random forest suggested that CQS-R, and specifically the Thinking/Feeling Compassionately subscale, is a primary predictor of symptomatology (including stress, anxiety, depression, and positive/negative affect) just after SCS self-coldness. This is significant, as most previous research suggested that symptoms such as depression and distress are mostly predicted by negative self-compassion items and subscales, such as self-coldness, rather than positive items and subscales such as self-warmth (Brenner et al., 2018; Körner et al., 2015; López et al., 2015). The case of the CQS-R Thinking/Feeling Compassionately, includes both positive and negative items, which adds nuance to our understanding and to the original self-compassion questionnaire (CQS). Scholarly, these results further strengthen the convergent and discriminant evidence supporting the validity of the revised questionnaires. From a clinical perspective, the significance of Thinking/Feeling Compassionately as a primary predictor of symptomatology and well-being underscores the importance of cultivating these skills within the context of cognitive-behavioral treatment as a means to reduce symptoms and enhance well-being. However, intervention studies and randomized controlled trials (RCTs) will be necessary to confirm these findings and explore their clinical implications more thoroughly.

The sixth and final objective of this study, as outlined at the outset, is to provide a more nuanced understanding of the parallels and disparities between self-compassion and compassion toward others, as well as their contributions to other psychological constructs such as symptoms and well-being. Findings from the current study bolster the differentiation between these two constructs, revealing discernible associations with variables related to the self versus others, while also indicating moderate levels of correlation between them. In addition, results from the suggested compassion profiles were consistent with previous findings (CQSO; Khoury et al., 2023) and recent evidence (Sahdra et al., 2023). This suggests that the three identified profiles (Low Compassion, Compassion for Others, and Integral Compassion) are stable and replicable, with Integral Compassion (i.e., compassion for self and others) presenting the highest benefits. This suggests that it is improbable to have self-compassion without also experiencing compassion toward others. One could speculate that compassion toward others serves as a foundational step necessary for cultivating self-compassion. This notion is intriguing, innovative, and carries important clinical and social implications, especially considering the predominant focus on self-compassion in Western psychology. However, such a hypothesis needs to be further tested in future experimental and longitudinal studies.

Moreover, results from using conditional random forest suggested that symptoms such as depression, as well as PANNAS, and well-being, can be predicted by a complex interaction between self-compassion (CQS-R) and compassion for others (CQO-R) scores captured by Conditional regression trees. For instance, concerning depressive symptoms, when individuals rate their level of Thinking/Feeling Compassionately toward themselves above a certain moderate level (e.g., above the mid-level of the scale), their levels of Thinking/Feeling Compassionately toward others and Connection with Others become significant predictors of their depression levels. Accordingly, individuals with higher levels of compassionate thinking/feeling toward others or stronger connections with others tend to exhibit lower depressive symptoms. Similar patterns were also observed in predicting negative affect. Conversely, for positive affect, Acting Compassionately toward others served as a complementary variable to Thinking/Feeling Compassionately toward oneself. Some of these similar patterns also emerged in predicting different dimensions of Ryff’s psychological well-being (Ryff, 1989), with Thinking/Feeling Compassionately toward others, Connection with Others, and Acting Compassionately toward Others being the primary complementary variables to Thinking/Feeling Compassionately toward oneself. Interestingly, Ryff’s Autonomy dimension (Ryff, 1989) was solely predicted by Acting Compassionately toward oneself, with higher self-compassionate actions predicting higher levels of autonomy. These results collectively underscore the central role of self-compassion in mental health and well-being and emphasize the potential of compassion for others as a complementary protective factor. Together, self-compassion and compassion for others contribute to the reduction of depressive symptoms and negative affect, while enhancing positive affect and psychological well-being.

In addition, in line with findings from the original versions of the compassion questionnaires (CQSO; Khoury et al., 2023), Thinking and Feeling compassionately remained integrated in both the revised self-compassion and compassion toward others questionnaire. Moreover, the revised questionnaires showed better differentiation from mindfulness, which was a central objective in developing the original questionnaires (CQSO; Khoury et al., 2023). Overall, the psychometric properties of the revised questionnaires (CQSO-R) were superior to the original versions (CQSO; Khoury et al., 2023).

In summary, the revised questionnaires demonstrated significant enhancements on both theoretical and empirical fronts. Theoretical advancements include broader coverage of underlying concepts and the inclusion of both positive and negative items. Empirical improvements encompassed enhanced psychometric properties, such as higher internal consistency, bolstered evidence of convergent and discriminant validity, and increased generalizability and utility.

### Limitations

The present study has several limitations. First, the participants’ sample was predominantly women, a common bias in compassion research, particularly when recruitment is primarily conducted through social media platforms such as Facebook. Despite this gender disparity and lack of invariance analysis, comparisons based on gender were conducted in line with the fourth objective of the study. Future research should reassess the reported gender differences and conduct a gender invariance analysis to ensure the validity of the findings across genders. Moreover, the majority of participants were English-speaking Canadians, reflecting the study’s locale, which potentially limits the generalizability of the CQSO-R to non-Canadian populations. Second, test-retest reliability over time was not assessed in this study. Similarly, the obtained sample presented a significant amount of missing data, which was handled under the assumption of Missing Completely at Random. Our split-half cross-validation, accompanied by the estimation of 95% confidence intervals, supported that despite the missingness, the sample behaved reasonably homogeneously. Nonetheless, potential biases arising from sample composition and missing data should be considered in future research. Third, the interplay between compassion for self and others, although investigated according to the sixth objective of the study, still requires further delineation (e.g., the potential need for the cultivation of compassion for others as a precursor to the cultivation of self-compassion) through experimental and longitudinal studies. Fourth, the clinical utility of the revised questionnaires was not evaluated in the current study. RCTs employing compassion training practices (e.g., Khoury et al., 2025) are needed to determine the efficacy and limitations of the CQSO-R in assessing changes following a compassion-focused intervention. Last, the survey was presented in a fixed order to streamline participant administration, resulting in higher attrition rates for later instruments compared to those at the survey’s outset, which could introduce biases into the obtained results based on nonrandom missingness. However, results from the computations of the internal consistencies of all the measures used in the study suggest very good values, regardless of their order of presentation to the participants. This indicates a high overall quality of the collected data. Overall, despite these limitations, the current study successfully achieved its six stated goals by revising and enhancing the newly developed CQSO, thereby facilitating their use with a wider population, ultimately increasing their potential impact.

### Implications

The CQSO represented a pioneering effort in conceptualizing compassion toward self and others as multifaceted sets of affective, cognitive, behavioral, and interpersonal skills and abilities, distinct from mindfulness. This novel approach held significant theoretical and practical implications. Theoretically, it provided a novel framework for examining compassion independently from mindfulness, expanding the scope of research in this area. Practically, by delineating compassion into various skills and abilities, each with differing levels of complexity and training requirements, the CQSO paved the way for integrating compassion training into existing cognitive-behavioral interventions and compassion/mindfulness-based programs. This not only enhances the comprehensiveness of such interventions but also opens avenues for tailored and targeted compassion-focused interventions.

The revised questionnaires capitalize on the strengths of their predecessors while systematically addressing their limitations, resulting in significantly enhanced theoretical and empirical robustness. In addition, the role of Thinking and Feeling Compassionately and the interplay between self-compassion and compassion for others have been further delineated with significant clinical implications. The full utility of the questionnaires awaits comprehensive exploration in forthcoming clinical trials.

## Supplemental Material

sj-docx-1-asm-10.1177_10731911251337185 – Supplemental material for Compassion Questionnaires Revised Supplemental material, sj-docx-1-asm-10.1177_10731911251337185 for Compassion Questionnaires Revised by Bassam Khoury and Rodrigo C. Vergara in Assessment

## Appendix A

### Compassion Questionnaire-Self Revised

*Instructions*: Listed below is a collection of statements about your everyday experiences. Please read each statement carefully, and using the scale below, please indicate how often you experience each of the following. Please answer according to what accurately reflects your experience rather than what you think your experience should be.

*(1) Almost Never*
*(2) Rarely*
*(3) Sometimes*
*(4) Often*
*(5) Almost Always*
  1. I feel bothered by my difficulties
  2. I have a hard time accepting care from others
  3. I tend to decline help from others
  4. When I fail, I think I am not good enough
  5. When I struggle, I try to soothe my suffering
  6. I take actions to ease my suffering during hard times
  7. During difficult times, I try to comfort myself
  8. I tend to refuse help from others
  9. When I struggle, I accept help from others
  10. I try to alleviate my suffering when I struggle
  11. I feel irritated by my shortcomings
  12. During hard times, I take actions to relieve my suffering
  13. When I struggle, I think I am a disappointment
  14. When I feel rejected, I think I am a failure
  15. When I feel bad, I try to console myself
  16. When I feel bad, I take actions to soothe myself
  17. When I have a hard time, I think it is my fault
  18. When I make a mistake, I judge myself harshly
  19. I tend to reject care from others
  20. When I make a mistake, I feel mad at myself
  21. During difficult times, I am open to receiving help from others
  22. I am patient with my shortcomings
  23. During difficult times, I lean on others
  24. I feel angry at myself when I struggle
  25. I hate myself when I fail
  26. I am forgiving of my failures and inadequacies
  27. I refrain from judging myself when making a mistake
  28. I have hard time accepting help from others
  29. I accept care from others when I need it
  30. When I fail, I think I am a total failure
  31. I can think I am good enough even after being rejected
  32. I try to help myself during difficult times
  33. I feel okay about myself after making a mistake
  34. I feel annoyed at myself when I do not understand something quickly
  35. I am open to receiving care when I need it
  36. When I feel rejected, I think I do not deserve love
  37. I feel disgusted by my errors
  38. I am kind to myself when I am struggling
  39. During difficult times, I try to relieve my pain

*Note. Each dimension is computed separately using the average of its items. The total score should be computed as the average of the three dimensions. R means the item is reverse scored.*

$$\begin{array}{c} 1.\;\mathrm{CQS}\left(\mathrm{Thinking}/\mathrm{Feeling}\;\mathrm{Compassionately}\right)\\ \;=(\mathrm{CQS}1R+\mathrm{CQS}4R+\mathrm{CQS}11R+\mathrm{CQS}13R+\\ \;\;\mathrm{CQS}14R+\mathrm{CQS}17R+\mathrm{CQS}18R+\mathrm{CQS}20R+\\ \;\mathrm{CQS}22+\mathrm{CQS}24R+\mathrm{CQS}25R+\mathrm{CQS}26+\\ \;\mathrm{CQS}27+\mathrm{CQS}30R+\mathrm{CQS}31+\mathrm{CQS}33+\\ \;\mathrm{CQSR}34R+\mathrm{CQSR}36R+\mathrm{CQSR}37R+\mathrm{CQS}38)/20 \end{array}$$

$$\begin{array}{c} 2.\;\mathrm{CQS}\left(\mathrm{Connection}\;\mathrm{with}\;\mathrm{Others}\right)=(\mathrm{CQS}2R+\mathrm{CQS}3R\\ +\mathrm{CQS}8R+\mathrm{CQS}9+\mathrm{CQS}19R+\mathrm{CQS}21+\\ \mathrm{CQS}23+\mathrm{CQS}28R+\mathrm{CQS}29+\mathrm{CQS}35)/10 \end{array}$$

$$\begin{array}{c} 3.\;\mathrm{CQS}(\mathrm{Acting}\;\mathrm{or}\;\mathrm{Intention}\;\mathrm{to}\;\mathrm{Act}\;\mathrm{Compassionately})\\ \;=(\mathrm{CQS}5+\mathrm{CQS}6+\mathrm{CQS}7+\mathrm{CQS}10+\\ \;\;\mathrm{CQS}12+\mathrm{CQS}15+\mathrm{CQS}16+\mathrm{CQS}39+\mathrm{CQS}32)/9 \end{array}$$

$$\begin{array}{l} \mathrm{CQS}\left(\mathrm{totalscore}\right)=(\mathrm{CQS}(\mathrm{Thinking}/\mathrm{Feeling}\;\mathrm{Compassionately})\\ +\mathrm{CQS}(\mathrm{Connection}\;\mathrm{with}\;\mathrm{Others})\\ +\mathrm{CQS}(\mathrm{Acting}\;\mathrm{or}\;\mathrm{Intention}\;\mathrm{to}\;\mathrm{Act}\;\mathrm{Compassionately}))/3 \end{array}$$

### Compassion Questionnaire-Others Revised

*Instructions*: Listed below is a collection of statements about your everyday experiences. Please read each statement carefully, and using the scale below, please indicate how often you experience each of the following. Please answer according to what accurately reflects your experience rather than what you think your experience should be.

*(1) Almost Never*
*(2) Rarely*
*(3) Sometimes*
*(4) Often*
*(5) Almost Always*
  1. Common suffering makes me feel closer to other people during hard times
  2. My suffering helps me connect with other people’s suffering
  3. I judge others ruthlessly when they make a mistake
  4. During difficult times, I try to relieve the pain of others
  5. I try to alleviate the suffering of others when they struggle
  6. During hard times, I take actions to reduce the pain of others
  7. During hard times, I take actions to relieve the suffering of others
  8. I can feel positively toward others even after making a mistake
  9. I feel annoyed at others when they don’t understand things quickly
  10. I take actions to ease the suffering of others when they are having a hard time
  11. I judge others harshly when they make a mistake
  12. When someone struggles, I try to soothe their suffering
  13. When someone is feeling bad, I try to soothe them
  14. I am nonjudgmental of other people’s failures
  15. When someone fails, I think they deserve it
  16. When someone has a hard time, I think it is their mistake
  17. My struggles allow me to understand the struggles of others
  18. I help others when they have a tough time
  19. When someone is feeling bad, I try to soothe their suffering
  20. I refrain from judging others when they make a mistake
  21. My pain helps me connect with other people’s pain
  22. I notice the commonalities between my suffering and the suffering of others
  23. During difficult times, I try to console others
  24. I try to comfort others when they are suffering
  25. I am accepting of other people’s mistakes
  26. My difficulties make it easier to understand other people’s difficulties
  27. I am very critical of other people’s flaws and inadequacies
  28. I am very forgiving of other people’s flaws and inadequacies
  29. I take care of others when they are in need
  30. When someone makes a mistake, I feel mad at them
  31. My struggles do not help me relate to other people’s struggles
  32. I try to help others when they have difficult times
  33. I feel irritated by the shortcomings of others

*Note. Each dimension is computed separately using the average of its items. The total score should be computed as the average of the three dimensions. R means the item is reverse scored.*

$$\begin{array}{c} 1\;\mathrm{CQO}\left(\mathrm{Thinking}/\mathrm{Feeling}\;\mathrm{Compassionately}\right)\\ \;\;\;\;=(\mathrm{CQO}3R+\mathrm{CQO}8+\mathrm{CQO}9R+\mathrm{CQO}11R+\\ \;\mathrm{CQO}14+\mathrm{CQO}15R+\mathrm{CQO}16R+\mathrm{CQO}20+\\ \;\mathrm{CQO}25+\mathrm{CQOR}27R+\mathrm{CQO}28+\mathrm{CQO}30R+\\ \;\mathrm{CQO}33R)/13 \end{array}$$

$$\begin{array}{c} 2\;\mathrm{CQO}\left(\mathrm{Connection}\;\mathrm{with}\;\mathrm{Others}\right)=(\mathrm{CQO}1+\mathrm{CQO}2\\ \;+\mathrm{CQO}17+\mathrm{CQO}21+\mathrm{CQO}22+\mathrm{CQO}26\\ \;+\mathrm{CQO}31R)/7 \end{array}$$

$$\begin{array}{c} \;3\;\mathrm{CQO}(\mathrm{Acting}\;\mathrm{or}\;\mathrm{Intention}\;\mathrm{to}\;\mathrm{Act}\;\mathrm{Compassionately})\\ \;=(\mathrm{CQO}4+\mathrm{CQO}5+\mathrm{CQO}6+\mathrm{CQO}7+\mathrm{CQO}10+\\ \;\;\mathrm{CQO}12+\mathrm{CQO}13+\mathrm{CQO}18+\mathrm{CQO}19+\mathrm{CQO}23+\\ \mathrm{CQO}24+\mathrm{CQO}29+\mathrm{CQO}32)/13 \end{array}$$

$$\begin{array}{l} \mathrm{CQO}\left(\mathrm{total}\;\mathrm{score}\right)=(\mathrm{CQO}(\mathrm{Thinking}/\mathrm{Feeling}\;\mathrm{Compassionately})\\ +\mathrm{CQO}(\mathrm{Connection}\;\mathrm{with}\;\mathrm{Others})\\ +\mathrm{CQO}(\mathrm{Acting}\;\mathrm{or}\;\mathrm{Intention}\;\mathrm{to}\;\mathrm{Act}\;\mathrm{Compassionately}))/3 \end{array}$$

## Appendix B

### Changes from the Original Version of the Questionnaires

In the final version of the Compassion Questionnaire-Self Revised (CQS-R) Thinking/Feeling Compassionately dimension, six positive items were added (all items were negatively worded in the original version), for example, CQS-R22, “I am patient with my shortcomings,” CQS-R26, “I am forgiving of my failures and inadequacies,” and CQS-R38, “I am kind to myself when I am struggling.” Negative items remained largely the same, but some were slightly modified for better clarity and results, for example, CQS18, “When I fail, I think I am stupid,” was reworded to CQS-R30, “When I fail, I think I am a total failure.” However, these changes were limited to a few items and remained minimal.

In the CQS-R Acting Compassionately dimension, the number of items increased significantly from three in the previous version to nine in the current one. Two of the three previous items were also mildly reworded for better clarity, for example, CQS4, “During difficult times, I take actions to feel better,” was reworded as CQS-R7, “During difficult times, I try to comfort myself.” However, all items in both versions remained positively worded, as from data, it appeared not useful to include negatively worded items when describing concrete actions to ease someone’s suffering without getting into other concepts/behaviors such as self-harm or self-punishment.

In the CQS-R Connecting with Others Compassionately dimension, four negatively worded items were added, such as CQS-R2, “I have a hard time accepting care from others,” allowing for a better balance between positively and negatively worded items, resulting in five of each (i.e., five positive and five negative items). Previous items remained unchanged in the revised version.

From another side, in the final version of the Compassion Questionnaire-Others Revised (CQO-R) Thinking/Feeling Compassionately, five positive items were added (all items were negatively worded in the original version), for example, CQO-R14, and “I am non-judgmental of other people’s failures,” CQSO-R28, “I am very forgiving of other people’s flaws and inadequacies.” Negative items remained largely the same, except two that were slightly modified for better clarity, for example, CQO10, “When someone fails, I think they are stupid,” which was reworded to CQO-R15, “When someone fails, I think they deserve it.”

In the CQO-R Acting Compassionately dimension, the number of items increased significantly from five in the previous version to 13 in the current one. Only one of the five previous items was also mildly reworded for better clarity, for example, CQO2, “When someone is feeling bad, I do whatever I can to make them feel better,” was reworded as CQO-R13, “When someone is feeling bad, I try to soothe them.” However, all items in both versions remained positively worded, as from data, it appeared not useful to include negatively worded items when describing concrete actions to ease someone else’s suffering.

In the CQO-R Connecting with Others Compassionately dimension, two positively and one negatively worded items were added such as CQO-R32, “My struggles do not help me relate to other people’s struggle,” allowing for a better coverage of the underlying concept and the inclusion of both positive and negative items.

## References

[bibr1-10731911251337185] Baer R. A. Smith G. T. Hopkins J. Krietemeyer J. Toney L. (2006). Using self-report assessment methods to explore facets of mindfulness. Assessment, 13(1), 27–45. 10.1177/1073191105283504 16443717

[bibr2-10731911251337185] Baião R. Gilbert P. McEwan K. Carvalho S. (2015). Forms of self-criticising/attacking & self-reassuring scale: Psychometric properties and normative study. Psychology and Psychotherapy: Theory, Research and Practice, 88(4), 438–452. 10.1111/papt.12049 25492845

[bibr3-10731911251337185] Bandalos D. L. Finney S. J. (2010). Factor analysis: Exploratory and confirmatory. In Hancock G. R. Mueller R. O. (Eds.), The reviewer’s guide to quantitative methods in the social sciences (pp. 93–114). Routledge.

[bibr4-10731911251337185] Beck J. S. (2011). Cognitive behavior therapy: Basics and beyond. Guilford press.

[bibr5-10731911251337185] Berking M. Whitley B. (2014). Affect regulation training. Springer.

[bibr6-10731911251337185] Boateng G. O. Neilands T. B. Frongillo E. A. Melgar-Quiñonez H. R. Young S. L. (2018). Best practices for developing and validating scales for health, social, and behavioral research: A primer. Frontiers in Public Health, 6(149), 1–18. 10.3389/fpubh.2018.00149 PMC6004510 29942800

[bibr7-10731911251337185] Bodie G. D. (2011). The active-empathic listening scale (AELS): Conceptualization and evidence of validity within the interpersonal domain. Communication Quarterly, 59(3), 277–295. 10.1080/01463373.2011.583495

[bibr8-10731911251337185] Brenner R. E. Vogel D. L. Lannin D. G. Engel K. E. Seidman A. J. Heath P. J. (2018). Do self-compassion and self-coldness distinctly relate to distress and well-being? A theoretical model of self-relating. Journal of Counseling Psychology, 65(3), 346–357. 10.1037/cou0000257 29672084

[bibr9-10731911251337185] Brown K. W. Ryan R. M. (2003). The benefits of being present: Mindfulness and its role in psychological well-being. Journal of Personality and Social Psychology, 84(4), 822–848. 10.1037/0022-3514.84.4.822 12703651

[bibr10-10731911251337185] Christov-Moore L. Simpson E. A. Coudé G. Grigaityte K. Iacoboni M. Ferrari P. F. (2014). Empathy: Gender effects in brain and behavior. Neuroscience and Biobehavioral Reviews, 46, 604–627. 10.1016/j.neubiorev.2014.09.001 25236781 PMC5110041

[bibr11-10731911251337185] Cohen S. Kamarck T. Mermelstein R. (1983). A global measure of perceived stress. Journal of Health and Social Behavior, 24(4), 385–396. 10.2307/2136404 6668417

[bibr12-10731911251337185] Coroiu A. Kwakkenbos L. Moran C. Thombs B. Albani C. Bourkas S. Zenger M. Brahler E. Körner A. (2018). Structural validation of the self-compassion scale with a German general population sample. PLoS ONE, 13(2), e0190771. 10.1371/journal.pone.0190771 PMC5800544 29408888

[bibr13-10731911251337185] Costa J. Marôco J. Pinto-Gouveia J. Ferreira C. Castilho P. (2016). Validation of the psychometric properties of the self-compassion scale. Testing the factorial validity and factorial invariance of the measure among borderline personality disorder, anxiety disorder, eating disorder and general populations. Clinical Psychology & Psychotherapy, 23(5), 460–468. 10.1002/cpp.1974 26289027

[bibr14-10731911251337185] Costello A. B. Osborne J. (2005). Best practices in exploratory factor analysis: Four recommendations for getting the most from your analysis. Practical Assessment, Research, and Evaluation, 10(1), 7. 10.7275/jyj1-4868

[bibr15-10731911251337185] Cronbach L. J. (1951). Coefficient alpha and the internal structure of tests. Psychometrika, 16(3), 297–334. 10.1007/BF02310555

[bibr16-10731911251337185] Crosby K. Skilton A. (1998). The Bodhicaryāvatāra. Oxford University Press.

[bibr17-10731911251337185] Dahl C. J. Lutz A. Davidson R. J. (2016). Cognitive processes are central in compassion meditation. Trends in Cognitive Sciences, 20(3), 161–162. 10.1016/j.tics.2015.12.005 26762882

[bibr18-10731911251337185] de Winter J. C. F. Dodou D. (2012). Factor recovery by principal axis factoring and maximum likelihood factor analysis as a function of factor pattern and sample size. Journal of Applied Statistics, 39(4), 695–710. 10.1080/02664763.2011.610445

[bibr19-10731911251337185] Di Fabio A. Saklofske D. H. (2021). The relationship of compassion and self-compassion with personality and emotional intelligence. Personality and Individual Differences, 169, 110109. 10.1016/j.paid.2020.110109 32394994 PMC7211602

[bibr20-10731911251337185] Diener E. Emmons R. A. Larsen R. J. Griffin S. (1985). The satisfaction with life scale. Journal of Personality Assessment, 49(1), 71–75. 10.1207/s15327752jpa4901_13 16367493

[bibr21-10731911251337185] Dunn T. J. Baguley T. Brunsden V. (2014). From alpha to omega: A practical solution to the pervasive problem of internal consistency estimation. British Journal of Psychology, 105(3), 399–412. 10.1111/bjop.12046 24844115

[bibr22-10731911251337185] El-Den S. Schneider C. Mirzaei A. Carter S. (2020). How to measure a latent construct: Psychometric principles for the development and validation of measurement instruments. International Journal of Pharmacy Practice, 28(4), 326–336. 10.1111/ijpp.12600 31944468

[bibr23-10731911251337185] Gerbing D. W. Hamilton J. G. (1996). Viability of exploratory factor analysis as a precursor to confirmatory factor analysis. Structural Equation Modeling: A Multidisciplinary Journal, 3(1), 62–72. 10.1080/10705519609540030

[bibr24-10731911251337185] Gilbert P. (2009). The compassionate mind: A new approach to life’s challenges. Constable and Robinson.

[bibr25-10731911251337185] Gilbert P. Catarino F. Duarte C. Matos M. Kolts R. Stubbs J. Ceresatto L. Duarte J. Pinto-Gouveia J. Basran J. (2017). The development of compassionate engagement and action scales for self and others. Journal of Compassionate Health Care, 4(1), 4. 10.1186/s40639-017-0033-3

[bibr26-10731911251337185] Gilbert P. McEwan K. Mitra R. Richter A. Franks L. Mills A. Bellew R. Gale C. (2009). An exploration of different types of positive affect in students and patients with bipolar disorder. Clinical Neuropsychiatry, 6, 135–143. http://hdl.handle.net/10545/622858

[bibr27-10731911251337185] Gu J. Baer R. Cavanagh K. Kuyken W. Strauss C. (2020). Development and psychometric properties of the Sussex-Oxford Compassion Scales (SOCS). Assessment, 27(1), 3–20. 10.1177/1073191119860911 31353931 PMC6906538

[bibr28-10731911251337185] Hayton J. C. Allen D. G. Scarpello V. (2004). Factor retention decisions in exploratory factor analysis: A tutorial on parallel analysis. Organizational Research Methods, 7(2), 191–205. 10.1177/1094428104263675

[bibr29-10731911251337185] Hothorn T. Bühlmann P. Dudoit S. Molinaro A. Van Der Laan M. J. (2005). Survival ensembles. Biostatistics, 7(3), 355–373. 10.1093/biostatistics/kxj011 16344280

[bibr30-10731911251337185] Hothorn T. Hornik K. Zeileis A. (2006). Unbiased recursive partitioning: A conditional inference framework. Journal of Computational and Graphical Statistics, 15(3), 651–674. 10.1198/106186006X133933

[bibr31-10731911251337185] Huysamen G. (2006). Coefficient alpha: Unnecessarily ambiguous; unduly ubiquitous. Journal of Industrial Psychology, 32(4), 34–40. 10.4102/sajip.v32i4.242

[bibr32-10731911251337185] Inwood E. Ferrari M. (2018). Mechanisms of change in the relationship between self-compassion, emotion regulation, and mental health: A systematic review. Applied Psychology: Health and Well-Being, 10(2), 215–235. 10.1111/aphw.12127 29673093

[bibr33-10731911251337185] Kanov J. M. Maitlis S. Worline M. C. Dutton J. E. Frost P. J. Lilius J. M. (2004). Compassion in organizational life. American Behavioral Scientist, 47(6), 808–827. 10.1177/0002764203260211

[bibr34-10731911251337185] Kelly A. C. Dupasquier J. (2016). Social safeness mediates the relationship between recalled parental warmth and the capacity for self-compassion and receiving compassion. Personality and Individual Differences, 89, 157–161. 10.1016/j.paid.2015.10.017

[bibr35-10731911251337185] Khoury B. (2019). Compassion: Embodied and embedded. Mindfulness, 10(11), 2363–2374. 10.1007/s12671-019-01211-w

[bibr36-10731911251337185] Khoury B. Dionne F. (2020). Les dimensions incarnée et interpersonnelle de la compassion. Annales Medico-Psychologiques, 178(1), 3–62. 10.1016/j.amp.2020.11.018

[bibr37-10731911251337185] Khoury B. Vergara R. C. (2024a). Compassion questionnaire for others (CQO). In Medvedev O. N. Krägeloh C. U. Siegert J. Singh N. N. (Eds.), Handbook of assessment in mindfulness research (pp. 1–14). Springer. 10.1007/978-3-030-77644-2_123-1

[bibr38-10731911251337185] Khoury B. Vergara R. C. (2024b). Compassion questionnaire for self (CQS). In Medvedev O. N. Krägeloh C. U. Siegert R. J. Singh N. N. (Eds.), Handbook of assessment in mindfulness research (pp. 1–15). Springer. 10.1007/978-3-030-77644-2_122-1

[bibr39-10731911251337185] Khoury B. Lesage M. Kasprzyk A. Sadowski I. Manova V. Benchimol-Elkaim B. Martins C. D. Rigas C. Rej S. (2025). Embodied and embedded mindfulness and compassion framework. Mindfulness. Advance online publication March 26, 2025. 10.1007/s12671-025-02561-4

[bibr40-10731911251337185] Khoury B. Vergara R. C. Spinelli C. (2023). Compassion questionnaires: Scales development and validation. Cognitive Therapy and Research, 47, 1006–1032. 10.1007/s10608-023-10416-2

[bibr41-10731911251337185] Körner A. Coroiu A. Copeland L. Gomez-Garibello C. Albani C. Zenger M. Brähler E. (2015). The role of self-compassion in buffering symptoms of depression in the general population. PLoS ONE, 10(10), e0136598. 10.1371/journal.pone.0136598 PMC4591980 26430893

[bibr42-10731911251337185] Lee R. M. Robbins S. B. (1995). Measuring belongingness: The social connectedness and the social assurance scales. Journal of Counseling Psychology, 42(2), 232–241. 10.1037/0022-0167.42.2.232

[bibr43-10731911251337185] López A. Sanderman R. Ranchor A. V. Schroevers M. J. (2018). Compassion for others and self-compassion: Levels, correlates, and relationship with psychological well-being. Mindfulness, 9(1), 325–331. 10.1007/s12671-017-0777-z 29387268 PMC5770484

[bibr44-10731911251337185] López A. Sanderman R. Smink A. Zhang Y. van Sonderen E. Ranchor A. Schroevers M. J. (2015). A reconsideration of the self-compassion scale’s total score: Self-compassion versus self-criticism. PLoS ONE, 10(7), e0132940. 10.1371/journal.pone.0132940 PMC4508060 26193654

[bibr45-10731911251337185] Lovibond S. Lovibond P. (1995). Manual for the depression anxiety stress scales (2nd ed.). Psychology Foundation.

[bibr46-10731911251337185] Ma J. Xiao Q. (2024). Relationship between self-compassion and compassion for others: The mediated effect of perceived social support and psychological resilience. Psychological Reports, Adavance online publication January 12, 2024. 10.1177/00332941241226906 38214161

[bibr47-10731911251337185] MacCallum R. C. Widaman K. F. Zhang S. Hong S. (1999). Sample size in factor analysis. Psychological Methods, 4(1), 84–99. 10.1037/1082-989X.4.1.84

[bibr48-10731911251337185] Maitreya A. Taye J. K. L. Gyamtso K. T. (2018). Buddha nature: The Mahayana Uttaratantra Shastra with commentary. Shambhala Publications.

[bibr49-10731911251337185] Messick S. (1995). Validity of psychological assessment: Validation of inferences from persons’ responses and performances as scientific inquiry into score meaning. American Psychologist, 50(9), 741–749. 10.1037/0003-066X.50.9.741

[bibr50-10731911251337185] Mills J. Wand T. Fraser J. A. (2018). Examining self-care, self-compassion and compassion for others: A cross-sectional survey of palliative care nurses and doctors. International Journal of Palliative Nursing, 24(1), 4–11. 10.12968/ijpn.2018.24.1.4 29368553

[bibr51-10731911251337185] Muris P. Petrocchi N. (2017). Protection or vulnerability? A meta-analysis of the relations between the positive and negative components of self-compassion and psychopathology. Clinical Psychology & Psychotherapy, 24(2), 373–383. 10.1002/cpp.2005 26891943

[bibr52-10731911251337185] Neff K. D. (2003a). The development and validation of a scale to measure self-compassion. Self and Identity, 2, 223–250. 10.1080/15298860309027

[bibr53-10731911251337185] Neff K. D. (2003b). Self-compassion: An alternative conceptualization of a healthy attitude toward oneself. Self and Identity, 2(2), 85–101. 10.1080/15298860309032

[bibr54-10731911251337185] Neff K. D. Germer C. K. (2013). A pilot study and randomized controlled trial of the mindful self-compassion program. Journal of Clinical Psychology, 69(1), 28–44. 10.1002/jclp.21923 23070875

[bibr55-10731911251337185] Orcan F. (2018). Exploratory and confirmatory factor analysis: Which one to use first? Journal of Measurement and Evaluation in Education and Psychology, 9(4), 414–421. 10.21031/epod.394323

[bibr56-10731911251337185] Park J. J. Long P. Choe N. H. Schallert D. L. (2018). The contribution of self-compassion and compassion to others to students’ emotions and project commitment when experiencing conflict in group projects. International Journal of Educational Research, 88, 20–30. 10.1016/j.ijer.2018.01.009

[bibr57-10731911251337185] Peters G. (2014). The alpha and the omega of scale reliability and validity: Why and how to abandon Cronbach’s Alpha. European Health Psychologist, 16(2), 56–69.

[bibr58-10731911251337185] Pommier E. Neff K. D. Tóth-Király I. (2020). The development and validation of the Compassion Scale. Assessment, 27(1), 21–39. 10.1177/1073191119874108 31516024

[bibr59-10731911251337185] Ponce F. P. Irribarra D. T. Vergés A. Arias V. B. (2022). Wording effects in assessment: Missing the trees for the forest. Multivariate Behavioral Research, 57(5), 718–734. 10.1080/00273171.2021.1925075 34048313

[bibr60-10731911251337185] Ponce F. P. Torres Irribarra D. Vergés A. Arias V. B. (2023). The ephemeral nature of wording effects. Journal of Personality and Social Psychology, 125(6), 1472–1494. 10.1037/pspp0000471 37384461

[bibr61-10731911251337185] Pratscher S. D. Rose A. J. Markovitz L. Bettencourt A. (2018). Interpersonal mindfulness: Investigating mindfulness in interpersonal interactions, co-rumination, and friendship quality. Mindfulness, 9(4), 1206–1215. 10.1007/s12671-017-0859-y

[bibr62-10731911251337185] R Core Team. (2021). R: A *language and environment for statistical computing*. R Foundation for Statistical Computing. https://www.R-project.org/

[bibr63-10731911251337185] Raîche G. Walls T. A. Magis D. Riopel M. Blais J.-G. (2013). Non-graphical solutions for Cattell’s scree test. Methodology: European Journal of Research Methods for the Behavioral and Social Sciences, 9(1), 23–29. 10.1027/1614-2241/a000051

[bibr64-10731911251337185] Reio T. G. Shuck B. (2015). Exploratory factor analysis: Implications for theory, research, and practice. Advances in Developing Human Resources, 17(1), 12–25. 10.1177/1523422314559804

[bibr65-10731911251337185] Ryff C. (1989). Happiness is everything, or is it? Explorations on the meaning of psychological well-being. Journal of Personality and Social Psychology, 57, 1069–1081.

[bibr66-10731911251337185] Sahdra B. K. Ciarrochi J. Fraser M. I. Yap K. Haller E. Hayes S. C. Hofmann S. G. Gloster A. T. (2023). The compassion balance: Understanding the interrelation of self- and other-compassion for optimal well-being. Mindfulness, 14(8), 1997–2013. 10.1007/s12671-023-02187-4

[bibr67-10731911251337185] Saputra D. M. Saputra D. Oswari L. D. (2020). Effect of distance metrics in determining K-value in K-means clustering using Elbow and Silhouette method.

[bibr68-10731911251337185] Schmitt T. A. (2011). Current methodological considerations in exploratory and confirmatory factor analysis. Journal of Psychoeducational Assessment, 29(4), 304–321. 10.1177/0734282911406653

[bibr69-10731911251337185] Shonin E. Van Gordon W. Griffiths M. D. (2014). The emerging role of Buddhism in clinical psychology: Toward effective integration. Psychology of Religion and Spirituality, 6(2), 123–137. 10.1037/a0035859

[bibr70-10731911251337185] Sijtsma K. (2009). On the use, the misuse, and the very limited usefulness of Cronbach’s alpha. Psychometrika, 74(1), 107–120. 10.1007/s11336-008-9101-0 20037639 PMC2792363

[bibr71-10731911251337185] Sinclair S. Kondejewski J. Raffin-Bouchal S. King-Shier K. M. Singh P. (2017). Can self-compassion promote healthcare provider well-being and compassionate care to others? Results of a systematic review. Applied Psychology: Health and Well-Being, 9(2), 168–206. 10.1111/aphw.12086 28393485

[bibr72-10731911251337185] Spreng R. N. McKinnon M. C. Mar R. A. Levine B. (2009). The Toronto empathy questionnaire: Scale development and initial validation of a factor-analytic solution to multiple empathy measures. Journal of Personality Assessment, 91(1), 62–71. 10.1080/00223890802484381 19085285 PMC2775495

[bibr73-10731911251337185] Strauss C. Lever Taylor B. Gu J. Kuyken W. Baer R. Jones F. Cavanagh K. (2016). What is compassion and how can we measure it? A review of definitions and measures. Clinical Psychology Review, 47, 15–27. 10.1016/j.cpr.2016.05.004 27267346

[bibr74-10731911251337185] Strobl C. Boulesteix A.-L. Kneib T. Augustin T. Zeileis A. (2008). Conditional variable importance for random forests. BMC Bioinformatics, 9(1), 307. 10.1186/1471-2105-9-307 18620558 PMC2491635

[bibr75-10731911251337185] Strobl C. Boulesteix A.-L. Zeileis A. Hothorn T. (2007). Bias in random forest variable importance measures: Illustrations, sources and a solution. BMC Bioinformatics, 8(1), 25. 10.1186/1471-2105-8-25 17254353 PMC1796903

[bibr76-10731911251337185] Tirch D. D. (2010). Mindfulness as a context for the cultivation of compassion. International Journal of Cognitive Therapy, 3(2), 113–123. 10.1521/ijct.2010.3.2.113

[bibr77-10731911251337185] Watson D. Clark L. A. Tellegen A. (1988). Development and validation of brief measures of positive and negative affect: The PANAS scales. Journal of Personality and Social Psychology, 54(6), 1063–1070. 10.1037/0022-3514.54.6.1063 3397865

[bibr78-10731911251337185] Wispé L. (1991). The psychology of sympathy. Plenum.

[bibr79-10731911251337185] Yang Y. Zhang M. Kou Y. (2016). Self-compassion and life satisfaction: The mediating role of hope. Personality and Individual Differences, 98, 91–95. 10.1016/j.paid.2016.03.086

[bibr80-10731911251337185] Yarnell L. M. Neff K. D. Davidson O. A. Mullarkey M. (2019). Gender differences in self-compassion: Examining the role of gender role orientation. Mindfulness, 10(6), 1136–1152. 10.1007/s12671-018-1066-1

[bibr81-10731911251337185] Yarnell L. M. Stafford R. E. Neff K. D. Reilly E. D. Knox M. C. Mullarkey M. (2015). Meta-analysis of gender differences in self-compassion. Self and Identity, 14(5), 499–520. 10.1080/15298868.2015.1029966

[bibr82-10731911251337185] Zeileis A. Hothorn T. Hornik K. (2008). Model-based recursive partitioning. Journal of Computational and Graphical Statistics, 17(2), 492–514. 10.1198/106186008X319331

[bibr83-10731911251337185] Zessin U. Dickhäuser O. Garbade S. (2015). The relationship between self-compassion and well-being: A meta-analysis. Applied Psychology: Health and Well-Being, 7(3), 340–364. 10.1111/aphw.12051 26311196

