# Supplementary material for: Compassion Questionnaires Revised: Scales Development and Validation
Source: Assessment. 2025 May 17;33(4):653–87. doi: 10.1177/10731911251337185 (PMC13153449; doi:10.1177/10731911251337185)
Supplement: sj-docx-1-asm-10.1177_10731911251337185 – Supplemental material for Compassion Questionnaires Revised [file sj-docx-1-asm-10.1177_10731911251337185.docx]

**Supplementary Material**

1. Items skewness
   1. *Methods*

During the construct validity assessment of the CQO, we identified both Heywood and Ultra-Heywood cases. There are several factors that may contribute to the occurrence of Heywood cases (Farooq, 2022). Based on our experience with the initial version of the CQO, we hypothesized that items exhibiting high skewness were likely contributing to this issue. It is important to note that skewed items are not inherently problematic, as they can enhance the sensitivity of the instrument by capturing the trait at different intensities across a broader range. However, this must be carefully balanced to ensure that the structure of the instrument is not compromised.

Moreover, it is crucial to recognize that skewness alone is not the sole factor influencing the emergence of Heywood cases. Items with low variance or those influenced by outliers can also lead to such issues (Farooq, 2022). Given the absence of a consensus on an ideal cutoff for removing items causing Heywood cases, we sought a balanced approach. While skewed items can contribute positively to the instrument, we adopted a criterion that addresses both high skewness and low variance, with a particular focus on distributions prone to outlier effects.

To refine this, we applied a criterion where 80% of responses for an item fell within the top (5 and 4) or bottom (1 and 2) Likert scale categories. This approach helps us identify items that exhibit severe skewness while also accounting for those with low variance, which may not be highly skewed but still exhibit undesirable distribution patterns.

To assess item skewness, we first visually inspected the distribution of each CQO-R item. Then, we examined the descriptive statistics for each item, including mean, standard deviation, median, minimum, maximum, skewness, and kurtosis. Items selected for removal were carefully analyzed based on these descriptive statistics to ensure they met the criteria for removal without unduly impacting the instrument’s validity.

- 1. *Results*

In general, asymmetry was observed in many of the CQO-R items. Figure S1 displays the item distributions ordered by median, where it is evident that some items show a concentration of scores within the 1 (5) and 2 (4) categories, with few observations at Likert score 3, and almost no observations at scores 5 (1) or 4 (2). Only a few items were evenly distributed across the five-point Likert scale, indicating that most CQO-R items exhibited at least some degree of skewness.

When reviewing the descriptive statistics of each CQO-R item, some showed skewness values exceeding 1, with means either below 2 or above 4 (Table S1). Table S1 highlights the removed items in bold, all of which displayed skewness near or above 1, with means outside the central range (below 2 or above 4) and standard deviations below 0.95. This criterion effectively identified highly skewed items, where the majority of responses fell into the extremes of the scale, indicative of ceiling or floor effects. Additionally, the criterion detected moderately skewed items with low variance.

Overall, the removal of items based on this criterion successfully prevented Heywood and Ultra-Heywood cases, while also preserving most items with moderate skewness. This approach ensures that the instrument maintains sensitivity to varying intensities (i.e., difficulty) of the measured trait

**Figure S1**. CQO-R items distribution ordered by median.


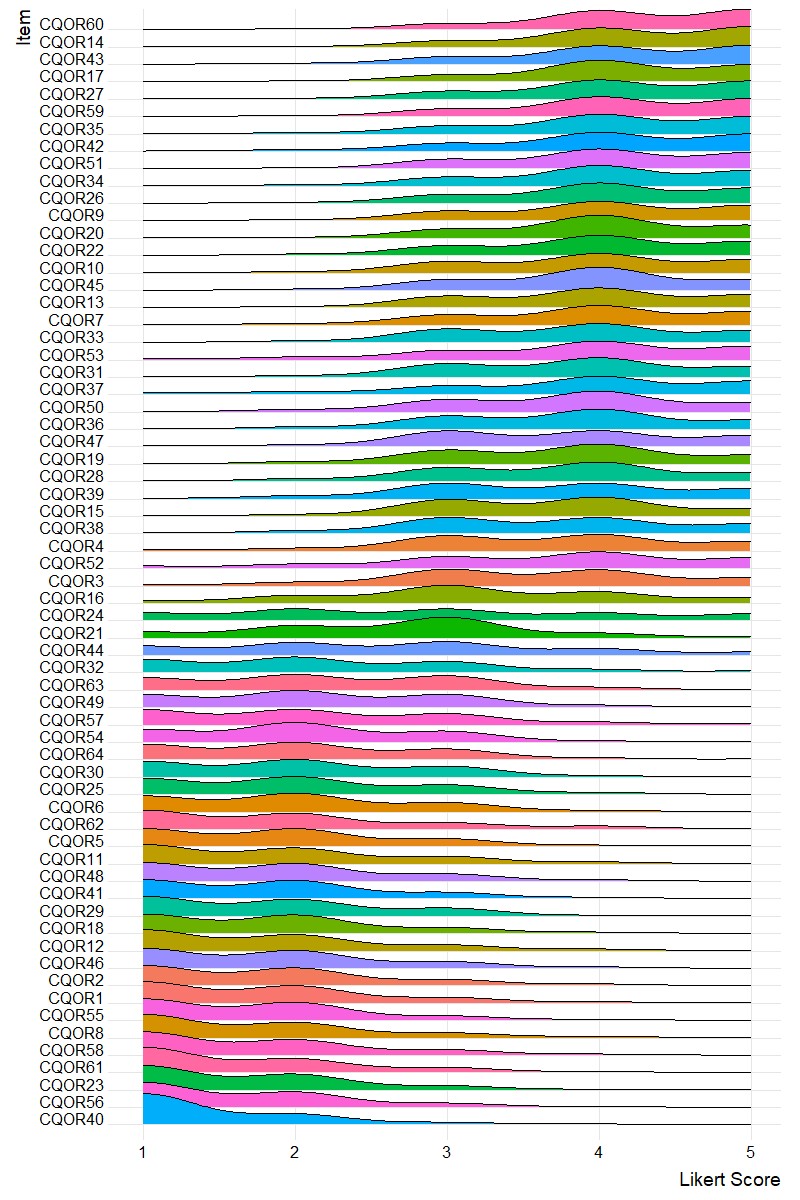


**Table S1.** Descriptive statistic from all CQOR items evaluated. In bold items that were removed by suspicion of contributing to Heywood or Ultra-Heywood cases due to skewness.

| *Item ID* | *n* | *mean* | *sd* | *median* | *min* | *max* | *skew* | *kurtosis* |
| --- | --- | --- | --- | --- | --- | --- | --- | --- |
| **1** | **912** | **1.76** | **0.85** | **2** | **1** | **5** | **1.10** | **1.03** |
| **2** | **912** | **1.80** | **0.87** | **2** | **1** | **5** | **0.97** | **0.56** |
| 3 | 912 | 3.54 | 0.99 | 4 | 1 | 5 | -0.39 | -0.07 |
| 4 | 912 | 3.65 | 0.99 | 4 | 1 | 5 | -0.48 | -0.02 |
| 5 | 912 | 1.92 | 0.95 | 2 | 1 | 5 | 1.03 | 0.94 |
| 6 | 912 | 1.99 | 0.93 | 2 | 1 | 5 | 0.88 | 0.67 |
| 7 | 912 | 3.92 | 0.94 | 4 | 1 | 5 | -0.82 | 0.64 |
| **8** | **912** | **1.71** | **0.88** | **1** | **1** | **5** | **1.25** | **1.26** |
| 9 | 912 | 4.04 | 0.87 | 4 | 1 | 5 | -0.74 | 0.46 |
| 10 | 912 | 3.94 | 0.88 | 4 | 1 | 5 | -0.61 | 0.25 |
| 11 | 912 | 1.91 | 0.98 | 2 | 1 | 5 | 1.02 | 0.62 |
| **12** | **912** | **1.81** | **0.93** | **2** | **1** | **5** | **1.15** | **0.99** |
| 13 | 912 | 3.93 | 0.88 | 4 | 1 | 5 | -0.64 | 0.43 |
| **14** | **912** | **4.24** | **0.81** | **4** | **1** | **5** | **-1.06** | **1.43** |
| 15 | 912 | 3.68 | 0.85 | 4 | 1 | 5 | -0.28 | 0.10 |
| 16 | 912 | 3.20 | 1.07 | 3 | 1 | 5 | -0.10 | -0.49 |
| **17** | **912** | **4.15** | **0.79** | **4** | **1** | **5** | **-0.83** | **0.85** |
| **18** | **912** | **1.82** | **0.88** | **2** | **1** | **5** | **1.10** | **1.16** |
| 19 | 912 | 3.74 | 0.88 | 4 | 1 | 5 | -0.41 | 0.02 |
| 20 | 912 | 4.01 | 0.81 | 4 | 1 | 5 | -0.71 | 0.85 |
| 21 | 912 | 2.65 | 0.97 | 3 | 1 | 5 | 0.13 | -0.09 |
| 22 | 912 | 4.00 | 0.86 | 4 | 1 | 5 | -0.63 | 0.16 |
| **23** | **912** | **1.65** | **0.84** | **1** | **1** | **5** | **1.45** | **2.36** |
| 24 | 912 | 2.87 | 1.31 | 3 | 1 | 5 | 0.18 | -1.06 |
| 25 | 912 | 1.99 | 0.92 | 2 | 1 | 5 | 0.72 | 0.08 |
| 26 | 912 | 4.05 | 0.85 | 4 | 1 | 5 | -0.77 | 0.64 |
| 27 | 912 | 4.15 | 0.83 | 4 | 1 | 5 | -0.85 | 0.69 |
| 28 | 912 | 3.72 | 0.91 | 4 | 1 | 5 | -0.45 | 0.03 |
| 29 | 912 | 1.83 | 0.87 | 2 | 1 | 5 | 0.91 | 0.59 |
| 30 | 912 | 1.99 | 0.89 | 2 | 1 | 5 | 0.59 | -0.16 |
| 31 | 912 | 3.83 | 0.88 | 4 | 1 | 5 | -0.44 | 0.05 |
| 32 | 912 | 2.24 | 1.09 | 2 | 1 | 5 | 0.68 | -0.15 |
| 33 | 912 | 3.87 | 0.87 | 4 | 1 | 5 | -0.39 | -0.18 |
| 34 | 912 | 4.07 | 0.83 | 4 | 1 | 5 | -0.65 | 0.19 |
| 35 | 912 | 4.12 | 0.84 | 4 | 1 | 5 | -0.78 | 0.41 |
| 36 | 912 | 3.75 | 0.89 | 4 | 1 | 5 | -0.48 | 0.12 |
| 37 | 912 | 3.82 | 1.07 | 4 | 1 | 5 | -0.85 | 0.19 |
| 38 | 912 | 3.67 | 0.96 | 4 | 1 | 5 | -0.34 | -0.20 |
| 39 | 912 | 3.68 | 0.97 | 4 | 1 | 5 | -0.34 | -0.29 |
| **40** | **912** | **1.45** | **0.77** | **1** | **1** | **5** | **2.00** | **4.32** |
| 41 | 912 | 1.85 | 0.89 | 2 | 1 | 5 | 1.04 | 0.96 |
| 42 | 912 | 4.09 | 0.88 | 4 | 1 | 5 | -0.81 | 0.35 |
| 43 | 912 | 4.16 | 0.85 | 4 | 1 | 5 | -0.90 | 0.66 |
| 44 | 912 | 2.61 | 1.18 | 3 | 1 | 5 | 0.31 | -0.73 |
| 45 | 912 | 3.94 | 0.78 | 4 | 1 | 5 | -0.41 | 0.01 |
| **46** | **912** | **1.81** | **0.86** | **2** | **1** | **5** | **0.95** | **0.61** |
| 47 | 912 | 3.74 | 0.96 | 4 | 1 | 5 | -0.42 | -0.07 |
| 48 | 912 | 1.87 | 0.89 | 2 | 1 | 5 | 0.93 | 0.58 |
| 49 | 912 | 2.17 | 0.96 | 2 | 1 | 5 | 0.52 | -0.13 |
| 50 | 912 | 3.78 | 0.88 | 4 | 1 | 5 | -0.52 | 0.16 |
| 51 | 912 | 4.07 | 0.85 | 4 | 1 | 5 | -0.75 | 0.56 |
| 52 | 912 | 3.61 | 1.11 | 4 | 1 | 5 | -0.60 | -0.27 |
| 53 | 912 | 3.87 | 1.01 | 4 | 1 | 5 | -0.82 | 0.32 |
| 54 | 912 | 2.07 | 0.89 | 2 | 1 | 5 | 0.67 | 0.39 |
| **55** | **912** | **1.73** | **0.84** | **2** | **1** | **5** | **1.23** | **1.61** |
| **56** | **912** | **1.62** | **0.80** | **1** | **1** | **5** | **1.31** | **1.58** |
| 57 | 912 | 2.12 | 1.03 | 2 | 1 | 5 | 0.68 | -0.10 |
| **58** | **912** | **1.70** | **0.85** | **1** | **1** | **5** | **1.20** | **1.22** |
| 59 | 912 | 4.14 | 0.84 | 4 | 1 | 5 | -0.92 | 0.96 |
| **60** | **912** | **4.24** | **0.80** | **4** | **1** | **5** | **-0.99** | **1.02** |
| **61** | **912** | **1.65** | **0.85** | **1** | **1** | **5** | **1.34** | **1.57** |
| 62 | 912 | 1.94 | 1.02 | 2 | 1 | 5 | 1.02 | 0.40 |
| 63 | 912 | 2.24 | 0.99 | 2 | 1 | 5 | 0.45 | -0.26 |
| 64 | 912 | 2.04 | 0.95 | 2 | 1 | 5 | 0.74 | 0.23 |

1. Redaction Effect
   1. *Methods*

CQO and CQS include both positive and negative items, which may introduce redaction effects, as reported for other instruments (Ponce et al., 2022; 2023). To assess whether the instrument exhibits redaction effects, we follow a similar approach to that proposed by Ponce et al. (2023). This procedure involves profiling item responses to determine if a subset of participants answers negative and positive items inconsistently. This inconsistency may be masked during internal consistency analysis, while our approach is more sensitive to redaction effects. Furthermore, this method can capture item answering patterns in sub-samples that might not be detected at the item level.

To profile item responses, we conducted a clustering analysis. The rationale is that if we cluster a highly consistent subscale, we should obtain profiles ranging from low to high scores. Inconsistent patterns would suggest a lack of consistency between the items. Specifically, for redaction effects concerning positive and negative items, when present, we expect to find at least three profiles. The first two correspond to consistent patterns of low and high scores, while the third profile shows an inversion of scores for negative vs. positive items (Ponce et al., 2023), indicating inconsistency within a subsample.

We performed k-means clustering with 25 random starts, exploring a 5-cluster solution. Each clustering analysis was conducted per subscale, using the items in each subscale to form the clusters. No scaling was performed, as all items use the same scale. The rationale behind exploring five clusters is to search for one profile per Likert score, based on the assumption that an ideally consistent instrument would produce similar scores across all items, assuming they have the same difficulty. Recognizing that these are strong assumptions, we also examined the number of potential profiles using the Silhouette Method. We always extracted five clusters unless the Silhouette method suggested more than five.

This revision improves clarity and readability while maintaining the technical accuracy of your original explanation.

- 1. *Results*

As shown in Table S2, the Silhouette Method never suggested more than five clusters. Consequently, we consistently used a five-cluster solution. There were notable variations in cluster sizes, likely due to the convenience sampling. Upon examining Figure S2, we found that, in general, the clusters divided the sample into five distinct levels of scores, as expected in the absence of redaction effects. However, closer inspection of item CQO53 in Figure S2D revealed an unusual pattern in cluster five, suggesting that this item was interpreted differently by a subgroup of participants. Specifically, for cluster five, CQO-R 53 received low scores, while the rest of the cluster showed medium-high scores, indicating inconsistency for this item within this subgroup. As seen in Figure S2D, CQO-R 53 was consistent with the other items in all the remaining clusters. To prevent potential consistency issues due to item interpretation, we decided to remove CQO-R 53, considering that if this population profile increases in future uses of the instrument, it could affect subscale internal consistency.

Other unusual patterns, such as those shown in Figure S2E, where CQOR-3 in cluster one contributed to a centroid between clusters three and four, were not deemed significant, as the item contributed to the creation of a middle score. In Figure S2B, cluster four exhibited a slight redaction effect, with reverse items showing lower scores than non-reversed items. However, this was not a complete reversal of scores, but rather a minor intensity difference that could not be distinguished from item difficulty. Despite these differences, the scores remained within the range of clusters one and two, and the centroid of cluster four aligned between clusters one and two. While these results suggest potential differences in interpretation, the overall score distribution remained appropriate. In summary, these analyses support the conclusion that the revised versions of CQO and CQS do not exhibit redaction effects.

**Table S2**. Clusters sizes obtained for each subscale of CQO-R and CQS-R, and Silhouette Method suggestions of clusters to obtain for each subscale.

| **Scale** | **Subscale** | **Cluster size** | | | | | **Silhouette Method** |
| --- | --- | --- | --- | --- | --- | --- | --- |
|  |  | **1** | **2** | **3** | **4** | **5** |  |
| CQSR | TFC | 259 | 180 | 251 | 136 | 285 | 2 |
|  | CO | 179 | 265 | 335 | 119 | 221 | 5 |
|  | AIC | 91 | 117 | 321 | 339 | 252 | 2 |
| CQOR | TFC | 283 | 212 | 246 | 125 | 78 | 2 |
|  | CO | 87 | 274 | 180 | 201 | 208 | 2 |
|  | AIC | 230 | 25 | 192 | 156 | 346 | 3 |

*Clusters names are arbitrary. They are correlative to Figure S2.


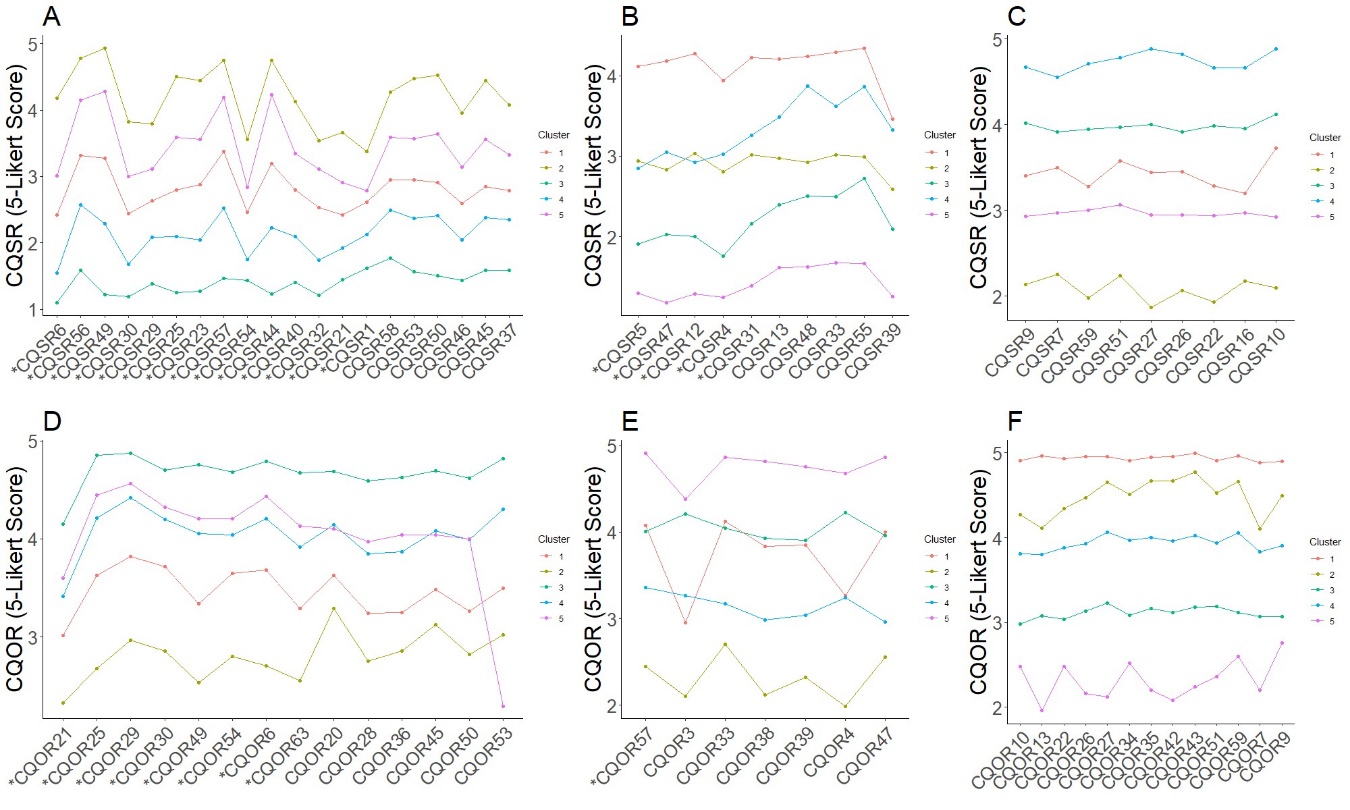


**Figure S2.** Cluster analysis results for each CQO-R and CQS-R subscale using items for clustering. Each point depicts the mean score of that item per cluster.

1. CART model interpretation

Conditional Regression Trees are a specific type of classification and regression trees (CART). All of these methods share the same basic rationale: recursively partitioning the sample to predict the dependent variable by identifying key cutoffs in the independent variables. Since this method is not frequently used, Figure S3 provides an example to guide the interpretation of the following figures.

CART results are typically presented in a decision tree format, as shown in Figure 3A. The tree begins with a root node (node 1), where the entire sample is intact and has not yet been partitioned. In node 1, the first partitioning occurs, and the reference variable for partitioning is depicted in the rectangle of node 1 (in Figure S3A, this is Variable 1). During this first partitioning, shown in Figure S1, Variable 1 is used to split the sample based on the criteria of >2 or ≤2. This means that the sample is divided at the cutoff of two, as illustrated in Figure S3B. In the tree, observations with Variable 1 > 2 move to a leaf or terminal node, indicating no further partitioning for this subset of data. Observations with Variable 1 ≤ 2 are then subjected to a second partitioning based on Variable 2, where the partitioning criterion is set at 3. As shown in Figure S3, this means splitting the group with Variable 1 ≤ 2 using the criterion of Variable 2 > 3 or ≤ 3. This second partitioning results in two additional leaves or terminal nodes.

Thus, the sample is divided into three groups through two partitioning steps. The second step, which splits a group derived from the first partition, exemplifies recursive partitioning. In conditional regression trees, each split is defined by the CART algorithm, which aims to minimize prediction error, and each split must be significant.


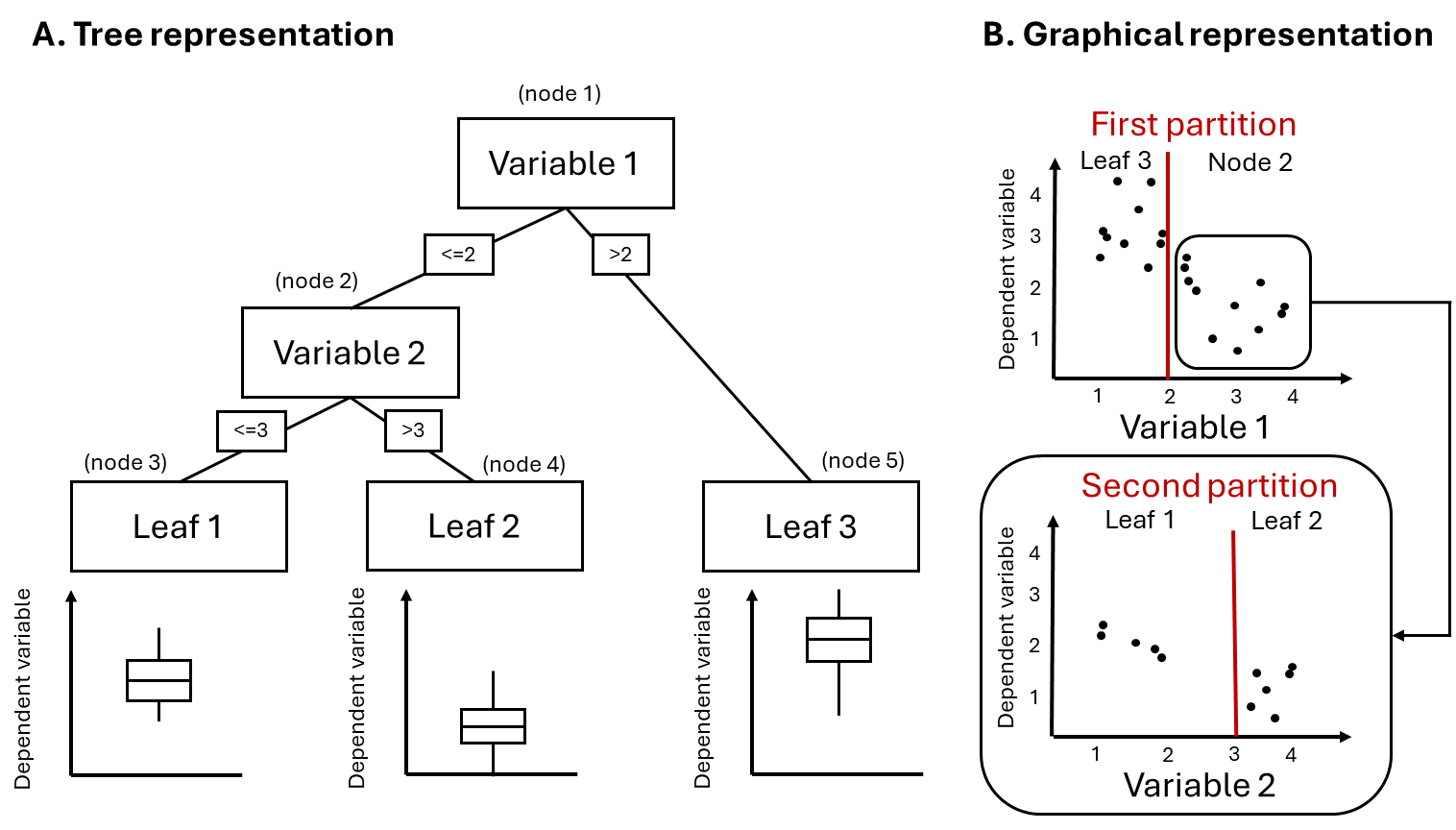


**Figure S3.** Schematic representation of a conditional regression tree, using a (A) tree representation and a (B) graphical representation.

Conditional Random Forest follows the same rationale as the individual trees but involves using a large number of trees (usually 1000). The core idea behind this method is that each tree "votes" for a prediction, and the final prediction is an aggregation of all the individual tree predictions. The "random" aspect of Random Forest comes from the random selection of variables that are used to construct each tree. Typically, a smaller subset of the total variables is randomly chosen for each tree. Because of this random selection, most trees in the forest will be different. This approach has strong predictive power with a good balance against overfitting.

However, interpreting the results of Random Forest models can be challenging due to their aggregating nature. One way to address this is by examining the variable importance. Variable importance measures the contribution of each variable based on the change in standard error when that variable is included in the different CART models that make up the forest.

Given that CART models are resilient to collinearity, they may still produce masking effects when two or more correlated predictors are used. Masking effects occur when predictors share variance and thus predictive information. In such cases, the CART algorithm selects the variable that provides the best prediction, potentially leaving other significant predictors out of the model. As a result, the reader may assume that absent variables are not important. However, due to masking effects, these variables could either be non-significant predictors or significant predictors that are correlated with variables already depicted in the CART diagram. To address this, the reader should consider the correlation matrix between the independent variables in the specific CART model. Additionally, examining the variable importance plots can offer insights into the relative importance of each predictor. Since Random Forests consist of 1000 trees, each using a random subset of dependent variables, we can confidently estimate the relative importance of each predictor, despite masking effects.

To account for the random nature of Random Forests, we performed 1000 Random Forest runs using 1000 different seeds (which help replicate the random selection process). This procedure allows us to calculate a confidence interval for the variable importance estimates. This approach can be considered as a form of variable screening, where we assess and compare the relative predictive value of many correlated predictors, ultimately identifying the most relevant variables for each dependent variable.

It is important to note that variable importance should always be interpreted in relative terms, not in absolute terms, as it depends on the error units (and is not standardized like squared R values). Moreover, a high relative importance does not necessarily imply that the predictor is statistically significant. For this reason, we first used Random Forest as a variable screening method and then confirmed the significance of the predictions through regression trees.

**References**

Farooq, R. (2022). Heywood cases: possible causes and solutions. *International Journal of Data Analysis Techniques and Strategies*, *14*(1), 79-88. <https://doi.org/10.1504/ijdats.2022.121506>

Ponce, F. P., Irribarra, D. T., Vergés, A., & Arias, V. B. (2022). Wording Effects in Assessment: Missing the Trees for the Forest. *Multivariate Behavioral Research*, *57*(5), 718-734. <https://doi.org/10.1080/00273171.2021.1925075>

Ponce, F. P., Torres Irribarra, D., Vergés, A., & Arias, V. B. (2023). The ephemeral nature of wording effects. *Journal of Personality and Social Psychology*, *125*(6), 1472-1494. <https://doi.org/10.1037/pspp0000471>
